# Genome mining to unravel potential metabolic pathways linked to gallium bioleaching ability of bacterial mine isolates

**DOI:** 10.3389/fmicb.2022.970147

**Published:** 2022-09-13

**Authors:** Ana Paula Chung, Romeu Francisco, Paula V. Morais, Rita Branco

**Affiliations:** Centre for Mechanical Engineering, Materials and Processes, Department of Life Sciences, University of Coimbra, Coimbra, Portugal

**Keywords:** mine isolates, genome analysis, metabolic features, gallium nitride (GaN), gallium arsenide (GaAs)

## Abstract

Gallium (Ga) is considered a high-tech Critical Metal, used in the manufacture of several microelectronic components containing either gallium arsenide (GaAs) or gallium nitride (GaN). The current high demand for this critical metal urges the development of effective recovery processes from secondary resources such as mine tailings or electronic recycling material. The importance of bioleaching as a biotechnological process to recover metals prompted this study, where an integrative approach combining experimental and genomic analysis was undertaken to identify potential mechanisms involved in bioleaching ability and strategies to cope with high metal(loid)s concentrations in five mine isolates. The Clusters of Orthologous Group (COG) annotation showed that the “amino acid transport and metabolism” [E] was the most predominant functional category in all genomes. In addition, the KEEG pathways analysis also showed predicted genes for the biosynthetic pathways of most amino acids, indicating that amino acids could have an important role in the Ga leaching mechanism. The presence of effective resistance mechanisms to Ga and arsenic (As) was particularly important in GaAs bioleaching batch assays, and might explain the divergence in bioleaching efficiency among the bacterial strains. *Rhodanobacter* sp. B2A1Ga4 and *Sphingomonas* sp. A2-49 with higher resistance, mainly to As, were the most efficient bioleaching strains under these conditions. In bioleaching assays using cell-free spent medium *Arthrobacter silviterrae* A2-55 with lower As resistance outperformed all the other stains. Overall, higher efficiency in Ga leaching was obtained in bioleaching assays using GaAs when compared to GaN.

## Introduction

Gallium (Ga) is a rare element with an abundance of about 16 ppm in the Earth’s crust (Moskalyk, 2003) and due to its semiconducting properties the industrial usage of this element has gained great interest (Gray et al., 2013). Nowadays, Ga is widely used in a variety of industrial applications and primarily in electronics. This high-tech metal is used in the manufacture of several microelectronic components containing either gallium arsenide (GaAs) or gallium nitride (GaN). Due to the technological advances and a modern society increasingly reliant on electronic devices, Ga has been classified as a critical metal, as being of high economic importance, and simultaneously subject to high supply risks (Hayes and McCullough, 2018; Girtan et al., 2021). In a circular economy concept, Ga and other critical metals can be recovered from secondary sources such as mine tailing and end-of-life electronic equipment (e-waste) (Hagelüken et al., 2016; Işıldar et al., 2019). E-wastes contain high-tech critical metals with a concentration higher than that present in the primary ores, which turns them a remarkable resource for metal recovery (Mishra et al., 2021).

Biotechnology processes such as the bioleaching that take advantage of microbial activity or metabolites produced by them, to solubilize valuable metals from secondary sources have developed into a successful and expanding area (Olson et al., 2003; Sedlakova-Kadukova et al., 2020). Bioleaching offers a more efficient and ecological biotechnology, with lower costs when compared to traditional physicochemical methods (Gao et al., 2020). A diverse group of bacteria found ubiquitously, or isolated from metal contaminated sites such as mines, is able to solubilize metals due to the production of metabolic products (Işıldar et al., 2019; Mishra et al., 2021). Several *Pseudomonas* species (Li et al., 2015; Potysz et al., 2016; Biswal et al., 2019), *Chromobacterium violaceum* (Li et al., 2015), and *Bacillus megaterium* (Motaghed et al., 2014), are cyanogenic bacteria able to bioleach e-waste, targeting valuable metals and the platinum group of metals (PGM), i.e., Au, Ag, Pt, Pd, Rh (Işıldar et al., 2019). These cyanogenic bacteria produce hydrogen cyanide and form water-soluble metal cyanide complexes by reacting with metals containing solids such as e-waste (Faramarzi et al., 2004; Brandl et al., 2008). Heterotrophic bacteria can also contribute to the bioleaching of critical metals through the production of organic acids solubilizing metals, namely, acetic acid, lactic acid, formic acid, oxalic acid, citric acid, succinic acid, and gluconic acid (Bosecker, 1997; Işıldar et al., 2019). Bioleaching of Rare Earth Elements (REE) from waste materials by biogenic gluconic acid was carried out by members of *Acinetobacter*, *Pseudomonas*, and *Gluconobacter* genera (Işıldar et al., 2019). Studies on bioleaching of Ga by heterotrophic bacteria are scanty, however, Maneesuwannarat and co-workers reported the bioleach of Ga from GaAs by *Cellulosimicrobium funkei* (Maneesuwannarat et al., 2016a) and from GaN by *Arthrobacter creatinolyticus* (Maneesuwannarat et al., 2016b), respectively.

Bioleaching bacteria show a biphasic response to metals in the environment (Pourhossein and Mousavi, 2018). They have the ability to solubilize and mobilize metals. However, when metal concentration increases beyond certain levels, it becomes toxic and has a negative effect on bioleaching efficiency. These bacteria have to rely on metal resistance mechanisms to survive, since high metal concentrations disrupt cellular function by damaging vital enzymatic functions, by directly affecting DNA structure, membrane lipids and proteins, and by disturbing ion balance (Chandrangsu et al., 2017; Igiri et al., 2018). Several bacterial are able to cope with metals and resist their toxicity using a diversity of strategies such as: modification of the metal redox state, metals precipitation or sorption to the cell surface, uptake and intracellular chelation, efflux mediated by specific transporters or the secretion of metal chelating agents to the environment (Srivastava and Kowshik, 2013).

The advances in high-quality and high-throughput sequencing technologies are massively increasing the number of bacterial genomes available, providing an opportunity to gain insights into several biological processes, such as the prediction of microbial interactions, the genetic diversity and the metal resistance mechanisms (Cárdenas et al., 2016; Zhang et al., 2018). Genome analysis can also improve our knowledge of bioleaching microorganisms by predicting their metabolic potential to mobilize and bioleach valuable metals.

In this work, the genomic sequence of five heterotrophic bacteria, isolated from different Portuguese mines was obtained (*Rugamonas* sp. A1-17, *Sphingomonas* sp. A2-49, *Arthrobacter silviterrae* A2-55, *Rhodanobacter* sp. B2A1Ga4, and *Undibacterium* sp. Jales W-56), and a comprehensive comparative genomic analysis based on several bioinformatics tools was performed to identify potential mechanisms involved in bioleaching ability and strategies to cope with high metal(loid)s concentrations. The ability of these five strains to bioleach Ga from GaAs and GaN was also determined in different experimental conditions.

## Materials and methods

### Bacterial strains isolation and growth

In a survey for bacteria with leaching ability, five bacterial strains showed the ability to mobilize Gallium (Ga) from GaAs and GaN and were selected for this study. These strains were isolated from different Portuguese mines and were deposited in the University of Coimbra Bacteria Culture Collection (UCCCB). *Rugamonas* sp. A1-17 (UCCCB48), *Sphingomonas* sp. A2-49 (UCCCB49), and *Arthrobacter* sp. A2-55 (UCCCB146), were isolated from the water of an uranium mine (Urgeiriça), *Rhodanobacter* sp. B2A1Ga4 (UCCCB 112) was isolated from sediments of a tungsten mine (Panasqueira) and *Undibacterium* sp. Jales W-56 (UCCCB147) was isolated from sediments from a gold mine (Jales). The bacterial strains were cultured in modified Reasoner’s 2A broth medium (mR2Ab), containing per liter: 0.25 g of yeast extract, 0.5 g of tryptone, 1.0 g of glucose, 0.3 g of K_2_HPO_4_, 0.024 g of MgSO_4_, and 0.3 g of sodium pyruvate. The pH of the medium was adjusted to pH 6.0. Cultures were grown at 25°C with orbital shaking at 140 rpm for 8 h (late exponential phase of growth), 24 h (stationary phase of growth) and 48 h (late stationary phase of growth), respectively.

### Genome sequencing, annotation, and strain identification

Total bacterial DNA extraction was performed using the E.Z.N.A.^®^ Bacterial DNA Kit (Omega Bio-Tek) according to manufacturer instructions. Libraries of total genomic DNA were prepared using Nextera XT Preparation Kit (Illumina, San Diego, CA, United States) following the manufacturer’s instructions. Libraries were purified using HighPrep PCR Clean-up beads (MagBio Genomics, Inc.). Fragment analyzer 5200 (Agilent NGS Fragment 1-6000 pb methods) was used to check the fragment size distribution and molarity of each library. Nine-picomolar libraries were sequenced on an Illumina MiSeq System with 2 × 300 bp chemistry (MiSeq Reagent Kit v3). Pairing, trimming, and assembly based on Bruijn graphs were performed using CLC Genomics Workbench v9.5.4 (Qiagen) using default parameters. Genome sequences were annotated using RAST server (Aziz et al., 2008) and the NCBI Prokaryotic Genome Annotation Pipeline (PGAP) (Tatusova et al., 2016). The draft genomes of *Rugamonas* sp. A1-17, *Sphingomonas* sp. A2-49, *Arthrobacter silviterrae* A2-55, and *Undibacterium* sp. Jales W-56 were deposited at DDBJ/ENA/GenBank under the accessions, JAJLPB000000000, JAJLPA000000000, JAJLOZ000000000, and JAJLQW000000000, respectively. The draft genome of *Rhodanobacter* sp. B2A1Ga-4 (JADBJR000000000) was already deposited at DDBJ/ENA/GenBank (Caldeira et al., 2021) and was used in this work. The taxonomic identification of the five mine isolates was based on the 16S rRNA gene by searching in the EzBioCloud Database the closest relative type strains (Yoon et al., 2017), and also by a genome-scale taxonomic analysis using the JSpeciesWS online services^1^ for calculation of the Average Nucleotide Identity based on the BLAST algorithm (ANIb) (Goris et al., 2007) and Tetranucleotide frequency correlation coefficient (TETRA) (Teeling et al., 2004) between the sequenced genomes and closely related type strains genomes. The type (strain) genome server (TYGS) (Meier-Kolthoff and Göker, 2019) was also used to confirm the taxonomic identities of the strains.

### Structural and functional analyses of bacterial genomes

The sequenced genomes were functionally annotated in terms of cluster of orthologous groups (COGs) using online eggNOG-mapper v2 (Cantalapiedra et al., 2021). Additionally, genomes were also annotated in terms of Kyoto Encyclopedia of Genes and Genomes (KEGG) orthology identifiers (KO) and mapped to KEGG pathways by the KEGG Automatic Annotation Server (KAAS) (Moriya et al., 2007), to gain insight about metabolic traits of the bacterial strains. The Venn diagram with shared and unique genes (COGs) among the 5 genomes was constructed using the Venn-Diagram free web tool of Bioinformatics and Evolutionary Genomics^2^.

The RAST Server (Aziz et al., 2008) and the COG functional annotation (Cantalapiedra et al., 2021) were used for identification of genes potentially involved in arsenic resistance and in iron transport. The resulting protein sequences were BLASTp searched against the non-redundant NCBI protein database for confirmation. Only those sequences that reported high hits to the correct functions were considered in our analyses and all of them were also checked in the NCBI Conserved Domains database.

### Minimum inhibitory concentration

The Minimum Inhibitory Concentration (MIC) for Ga and arsenite [As(III)] was determined in 96 multiwell plates using the standard broth micro dilution method in mR2Ab medium. The Ga concentration in the assays ranged between 10 and 0.125 mM and As(III) concentration ranged between 2.0 and 0.125 mM. Metal stock solutions were prepared in a concentration of 0.2 M gallium (III) nitrate (GaN_3_O_9_, Alfa Aesar) and 0.5 M sodium arsenite [NaAsO_2_, Merck)] and were sterilized by filtration. The assays were performed in duplicate to ensure the reproducibility of the experiments. Bacterial growths were analyzed after 48 h of incubation at 25°C. MIC values were the lowest concentration of each tested metal that inhibited growth of the microorganism after the incubation time in the appropriate growth conditions (Andrews, 2002). The inhibition of growth was considered as the absence of difference between the assay and its respective non-inoculated control, in terms of absorbance.

### Bioleaching experiments

The bioleaching experiments were performed in 20 ml of leaching medium containing either 10 mg of GaAs (Alfa Aesar) manually ground in a mortar, or 10 mg of GaN powder (Sigma Aldrich), at 25°C and 150 rpm in an orbital shaking incubator for 21 days. Three different types of bioleaching experiments were performed: (i) batch growth experiments, where the different strains were inoculated into mR2Ab medium (leaching medium) with an initial OD _600_ of 0.1; (ii) pre cultivated bacteria cultures at different growth phases (late exponential, 8 h; stationary, 24 h, and late stationary, 48 h) were used as leaching medium; and (iii) cell-free spent medium of cultures from stationary (24 h) and late stationary (48 h) phases of growth, which were obtained by centrifugation (9,000 rpm, 15 min.), followed by filtering through 0.2 μm filters, were used as leaching medium. Control experiments with mR2Ab medium without bacteria and in presence of GaAs or GaN were run in parallel and in the same conditions. All bioleaching experiments were run in duplicate or triplicate.

### Analytical methods

The pH was monitored at the beginning of each bioleach experiment and every 7 days until the end of the bioleaching period. pH variations were measured in a pH^®^ meter, pHenomenal pH 1100L (VWR Chemicals). Soluble Ga amount in liquid medium was quantified spectrophotometrically by a modification of the bromopyrogallol red (BPR) method (Huang et al., 1997), optimized for Ga. Briefly, samples were prepared by adding 200 μl of buffer solution (1.25 ml of 0.2 M KCl; 2.65 ml of 0.2 M HCL in 50 ml deionized water), 70 μl of 0.2% SDS (sodium dodecyl sulfate), 100 μl of leaching medium and 200 μl of 0.01% BPR in deionized water to make up a final volume of 1 ml. Calibration curve was prepared using standard Ga concentrations from 0 to 300 μM diluted from a gallium(III) nitrate (GaN_3_O_9_, Alfa Aesar) stock solution (1 mM). Samples were quantified spectrophotometrically at 540 nm.

### Statistical analysis

The Ga leaching capacities between all strains, incubation times, mineral substrate (GaAs, GaN) and growth phases of the biochemically active cells/cell-free spent media were analyzed by performing a multifactorial Permutational Multivariate Analysis of Variance (PERMANOVA) in order to determine significant differences between the factors analyzed (Bray-Curtis; Monte-Carlo test; PRIMER6 (v6.1.13) and PERMANOVA + (v1.0.3), PRIMER-E Ltd). Significant differences of leaching abilities between assays and controls were evaluated with a one-way analysis of variance (ANOVA) followed by Dunnett’s multiple comparison test using the software GraphPad Prism version 6.00 for Windows (GraphPad Software, San Diego, CA, United States)^3^.

## Results

### Taxonomic identification and genomic features of the assembled bacterial genomes

The taxonomic identification of the bacterial strains based on the 16S rRNA gene showed that the isolates from Urgeiriça mine A1-17, A2-49, and A2-55, were closely related to *Rugamonas aquatica* FT29W*^T^* with 98.41% of sequence similarity, *Sphingomonas aquatilis* JSS7*^T^* with 98.65% sequence similarity and *Arthrobacter silviterrae* KIS14-16*^T^* with 99.72% sequence similarity, respectively (Table 1). Strain Jales W-56 showed the highest 16S rRNA sequence similarity (97.34%) with *Undibacterium jejuense* JS4-4*^T^*. Strain B2A1Ga4 was already identified as a *Rhodanobacter* sp. (Caldeira et al., 2021) having as closest relative *Rhodanobacter thiooxydans* LCS2*^T^* with 98.98% of sequence similarity (Table 1). However, the values below the thresholds of 95.0% for ANIb and 0.989 for TETRA between the sequenced genomes and their closely related type strains genomes strongly indicates that they belong to different species (Supplementary Data 1). The only exception was obtained with strain A2-55, which have values of ANIb and TETRA with *Arthrobacter silviterrae* KIS14-16*^T^* above the threshold of 95.0% and 0.999, respectively, suggesting its inclusion in this species (Supplementary results). These results were further supported by the TYGS analysis, in particular by the digital DNA-DNA hybridization (dDDH) value of 80.3% between the genomes of strain A2-55 and *Arthrobacter silviterrae* KIS14-16*^T^*, higher than the threshold of 70.0% used to delineate species. All the other sequenced genomes showed dDDH values below the threshold 70.0% with their selected type strains genomes (Supplementary Data 2).

**TABLE 1:** Isolation local and genomic features of the mine bacterial strains.

Bacteria strain	*Rugamonas* sp. A1-17	*Sphingomonas* sp. A2-49	*A. silviterrae* A2-55	*Rhodanobacter* sp. B2A1Ga4	*Undibacterium* sp. JalesW-56
**Isolation local**	Urgeiriça mine (40°30’49.136 N, 7°53’40.311 W)	Urgeiriça mine (40°30’49.136 N, 7°53’40.311 W)	Urgeiriça mine (40°30’49.136 N, 7°53’40.311 W)	Panasqueira mine 40°9’4.237 N, 7°44’35.289 W)	Jales mine (41°27’50.796 N, 7°35’20.209 W)
**Closest relative (16S rRNA gene)**	*Rugamonas aquatica* FT29W^T^ (98.41%)	*Sphingomonas aquatilis* JSS7*^T^* (98.65%)	*Arthrobacter silviterrae* KIS14-16^T^ (99.72%)	*Rhodanobacter thiooxydans* LCS2^T^ (98.98%)	*Undibacterium jejuense* JS4-4^T^ (97.34%)
**Genome status**	Draft (JAJLPB000000000) (This study)	Draft (JAJLPA000000000) (This study)	Draft (AJLOZ000000000) (This study)	Draft (JADBJR000000000) (Caldeira et al., 2021)	Draft (JAJLQW000000000) (This study)
**Genome size (Mb)**	7.40	3.93	4.40	3.85	4.19
**Number of contigs**	86	57	161	10	23
**GC content (%)**	64.4	68.4	65.7	66.7	52.4
**RNAs genes**	76	51	65	56	49
**Coverage**	86.7	160.3	93.7	23.8	177.3
**N50**	367,641	131,993	47,181	514,946	569,399
**L50**	7	8	31	2	3
**Number of subsystems**	337	289	283	290	289
**Number of coding Sequences (CDS)**	6885	3877	4251	3580	4099

N50 is the sequence length of the shortest contig at 50% of the total genome length. L50 is the count of smallest number of contigs whose length sum makes up half of genome size.

The genome features of the four genomes obtained in this study by Illumina sequencing, as well as the genome features of *Rhodanobacter* sp. B2A1Ga4 previously sequenced (Caldeira et al., 2021) are summarized in Table 1. *Rugamonas* sp. A1-17 has a larger genome with 7.4 Mb compared to the other genomes which sized between 3.85 and 4.40 Mb. The *Undibacterium* Jales W-56 genome has the lowest G + C content (52.4%); all the other genomes have G + C contents higher than 64% (Table 1).

### Comparative genome analysis

#### Analysis of orthologous genes

**FIGURE 1:**
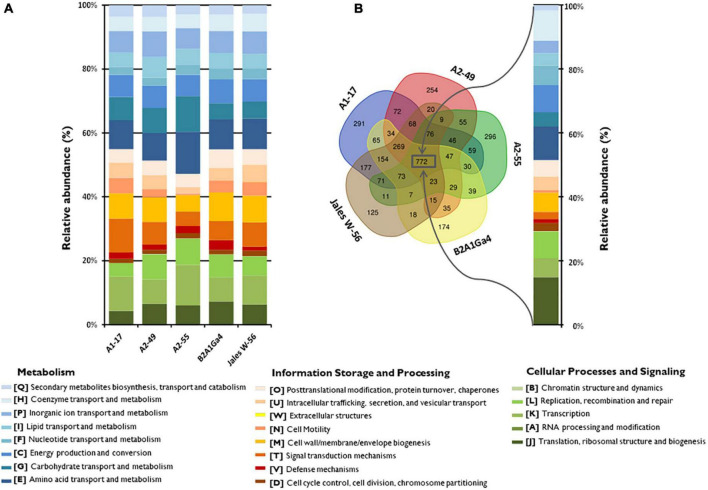
Distribution and comparison of COG functional categories in the analyzed genomes. **(A)** Relative abundances (%) of the 21 different COG functional categories in each genome according to three broad functional groups (Metabolism; Information Storage and Processing; Cellular Processes and Signaling). **(B)** Venn diagram showing the shared and unique gene clusters (COGs) among the genomes and the relative abundances (%) of the shared COG categories.

The Clusters of Orthologous Group (COG) annotation of the five genomes showed that 78.5 to 83.2% of the coding sequences (CDS) were matched to putative proteins with known functions and assigned to 21 of the COG categories (Figure 1A). The most abundant COG broad functional category in all genomes was “Metabolism” with 45.0 to 52.8% of genes involved in this function. Among this COG category, the “amino acid transport and metabolism” [E] was the most predominant functional category (8.5–13.2%), followed by “carbohydrate transport and metabolism” [G] (4.4 to 11.2%) and “energy production and conversion” [C] (6.7 to 8.5%), which were also well-represented functional categories (Figure 1A). The other two COG broad functional categories “Information Storage and Processing” represented about 20.2 to 35.6% and “Cellular Process and Signaling” represented 19.2 to 29.3% of the COG categories, respectively (Figure 1A). A comparative analysis of the genomes showed that 772 orthologous genes were shared by all the strains (Figure 1B). The COG functional categories determined for these shared genes were very similar to those assigned for each individual genome. The “amino acid transport and metabolism” [E] was the most predominant functional category (10.4%) among the “Metabolism” function. However, the shared genes were enriched in the functional category “translation, ribosomal structure and biogenesis” [J] representing 14.9%, while in individual genomes this category represented only 4.2 to 7.2% (Figures 1A,B).

#### Kyoto encyclopedia of genes and genomes pathway enrichment analysis

The metabolic potential of the genomes under study was also investigated by functional annotation and pathway mapping in the KEGG Automatic Annotation Server (KAAS). In all genomes, the majority of the genes was related to metabolism pathways (64.0–71.6%), and the gene families related to carbohydrate metabolism (15.3–19.3%) and amino acid metabolism (13.0–16.4%) were the most abundant among the metabolic genes (Supplementary Data 3). These results corroborate the ones obtained by the COG functional categories distributions. Since amino acids metabolism was predominant in these bacterial strains the biosynthetic pathways of each amino acid were further characterized and compared among these organisms. KEEG modules for the biosynthesis of amino acids threonine (M00018), tryptophan (M00023), arginine (M00844, M00845), lysine (M00016), proline (M00015), valine (M00019), isoleucine (M00019, M00570), leucine (M00432), and also the biosynthesis pathway for chorismate (M00022) the precursor of aromatic amino acids were present in all genomes (Table 2). None of the strains had the complete pathway for the biosynthesis of methionine from aspartate (M00017). Although the biosynthesis of glutamate, aspartate, asparagine and alanine were not represented in KEEG modules since they are derived from intermediates of central metabolism in short pathways, most of the strains had the potential to synthesize these amino acids. Exceptions were observed with strain *Sphingomonas* sp. A2-49 where the pyruvate to alanine conversion pathway was absent and strain *Undibacterium* sp. Jales W-56 where the oxaloacetate conversion pathway to aspartate is also absent. Glutamine and glycine conversion pathways from glutamate and threonine, respectively, were also present in all genomes.

**TABLE 2:** KEEG pathways and modules for amino acids biosynthesis and metabolism determined by KAAS (KEGG Automatic Annotation Server).

KEEG pathways	KEEG modules	*Rugamonas* sp. A1-17	*Sphingomonas* sp. A2-49	*A. silviterrae* A2-55	*Rhodanobacter* sp. Ga-4	*Undibacterium* sp. Jales W-56
**KO00260** Glycine, serine and threonine Metabolism	**M00020** Serine biosynthesis, glycerate-3P => serine	**√**	**√**	**√**	Incomplete	**√**
	**M00018** Threonine biosynthesis, aspartate => homoserine => threonine	**√**	**√**	**√**	**√**	**√**
**KO00270** Cysteine and methionine Metabolism	**M00021** Cysteine biosynthesis, serine => cysteine	**√**	**√**	**√**	Incomplete	Incomplete
	**M00338** Cysteine biosynthesis, homocysteine + serine => cysteine	**√**	**X**	**X**	**√**	**X**
	**M00017** Methionine biosynthesis, aspartate => homoserine =>methionine	Incomplete	Incomplete	Incomplete	Incomplete	Incomplete
**KO00400** Phenylalanine, tyrosine, and tryptophan biosynthesis	**M00022** Shikimate pathway, phosphoenolpyruvate + erythrose-4P => chorismate	**√**	**√**	**√**	**√**	**√**
	**M00023** Tryptophan biosynthesis, chorismate => tryptophan	**√**	**√**	**√**	**√**	**√**
	**M00024** Phenylalanine biosynthesis, chorismate => phenylpyruvate=>phenylalanine	**√**	Incomplete	Incomplete	**√**	**√**
	**M00025** Tyrosine biosynthesis, chorismate => HPP (4-Hydroxyphenylpyruvate) => tyrosine	**√**	Incomplete	Incomplete	Incomplete	**√**
**KO00220** Arginine biosynthesis	**M00028** Ornithine biosynthesis, glutamate => ornithine	**√**	**√**	**√**	Incomplete	**√**
	**M00844** Arginine biosynthesis, ornithine => arginine	**√**	**√**	**√**	Incomplete	**√**
	**M00845** Arginine biosynthesis, glutamate => acetylcitrulline => arginine	**X**	**X**	**X**	**√**	**X**
**KO00340** Histidine metabolism	**M00026** Histidine biosynthesis, PRPP (Phosphoribosyl Pyrophosphate) => histidine	Incomplete	**√**	**√**	**√**	Incomplete
**KO00300** Lysine biosynthesis	**M00016** Lysine biosynthesis, succinyl-DAP pathway, aspartate => lysine	**√**	**√**	**√**	**√**	**√**
**KO00330** Arginine and proline metabolism	**M00015** Proline biosynthesis, glutamate => proline	**√**	**√**	**√**	**√**	**√**
**KO00290** Valine, leucine, and isoleucine biosynthesis	**M00019** Valine/isoleucine biosynthesis, pyruvate => valine/2-oxobutanoate => isoleucine	**√**	**√**	**√**	**√**	**√**
	**M00570** Isoleucine biosynthesis, threonine => 2-oxobutanoate => isoleucine	**√**	**√**	**√**	**√**	**√**
	**M00432** Leucine biosynthesis, 2-oxoisovalerate => 2-oxoisocaproate	**√**	**√**	**√**	**√**	**√**

**√**, Module present and complete; **X**, module not present; Incomplete, module without one or two enzymes.

#### Arsenic resistance gene analysis

**FIGURE 2:**
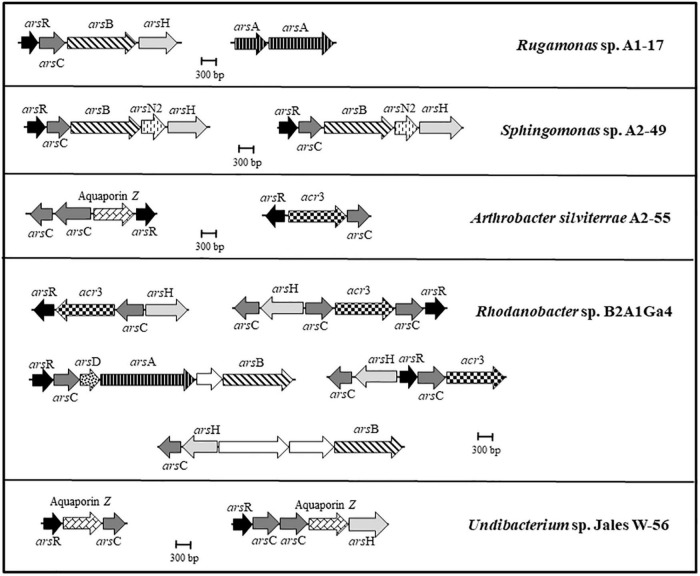
Genomic organization of the *ars* gene clusters in the analyzed genomes. Arrows represent different genes, and homologous genes are represented by the same color and pattern, except for the white arrows that represent genes identified as not being related to arsenic resistance.

The five genomes were analyzed to identify genes involved in arsenic resistance. The genomes of *A. silviterrae* A2-55 and *Undibacterium* sp. Jales W-56 showed simpler *ars* gene clusters, with an *arsR* gene encoding a metalloregulatory protein, an *acr3* or aquaporin Z gene encoding an arsenite efflux pump and an *arsC* gene encoding an enzyme able of reducing arsenate to arsenite (Figure 2). The arsenic gene cluster *arsRCBH* was identified in the genomes of *Rugamonas* sp. A1-17 and *Sphingomonas* sp. A2-49. This late strain was the only one to have *arsN2* genes in the *ars* gene clusters, however, the precise function of this gene has not yet been elucidated (Figure 2). The genome of *Rhodanobacter* sp. B2A1Ga4 showed a higher number and more complex *ars* gene clusters with a wide variety of gene configurations involved in arsenic resistance. This strain is the only one to present an *arsRCDAB* cluster, that besides the *arsR* and *arsC*, have *arsD*, *arsA*, and *arsB* genes adjacent, encoding an ArsA ATPase, an ArsD chaperone, and an arsenite efflux pump (Figure 2).

#### Iron transport systems

The mechanisms involved in Ga resistance are not well known, however, iron transport systems have an important role in this situation. The sequenced bacterial genomes showed the presence of iron uptake systems for both ferrous and ferric forms of elemental iron. In the genome of *Rugamonas* sp. A1-17 it was identified a larger number of putative outer membrane receptors for high-affinity uptake of siderophore-bound Fe^3+^ complexes when compared to the other Gram-negative bacterial genomes (*Sphingomonas* sp. A2-49, *Rhodanobacter* sp. B2A1Ga4, and *Undibacterium* sp. Jales W-56) (Table 3). The transport of iron-siderophore complexes through the outer membrane requires energy provided by the energy-transducing protein complex composed by TonB protein and the accessory proteins ExbB and ExbD (TonB-ExbB-ExbD). TonB proteins were predicted in all Gram-negative genomes and were present in a high number in the genomes of *Rugamonas* sp. A1-17 and *Undibacterium* sp. Jales W-56 (Table 3). Moreover, accessory proteins ExbB and ExbD were also present in all of these genomes, and in strains *Rugamonas* sp. A1-17, *Rhodanobacter* sp. B2A1Ga4, and *Undibacterium* sp. Jales W-56, in more than one set of ExbB-ExbD protein complex. *A. silviterrae* A2-55 as Gram-positive bacteria lacks an outer membrane and requires neither outer membrane receptors nor TonB-ExbB-ExbD systems. This Gram-positive strain has diverse ABC transporters analogous to those of the Gram-negative to transport iron-siderophore complexes across the cell membrane (Table 3). Among the Gram-negative bacteria, strains *Rugamonas* sp. A1-17 and *Undibacterium* sp. Jales W-56 were the only ones that presented putative periplasmic ABC transporters (AfuA: ABC-type Fe^3+^ transport system, FepB: ABC-type Fe^3+^-hydroxamate transport system) that are able to shuttle iron-siderophore complexes from outer- membrane receptors to the cytoplasmic membrane ABC transporters (FbpB: ABC-type Fe^3+^ transport system, FepC: ABC-type cobalamin/Fe^3+^-siderophores transport system, and FepD: ABC-type Fe^3+^-siderophore transport system). The genome of *Rhodanobacter* sp. B2A1Ga4 only showed a putative periplasmic ABC transporter and the genome of *Sphingomonas* sp. A2-49 only showed cytoplasmic membrane ABC transporters (Table 3). A putative iron export permease protein (FetB: ABC-type iron transport system) was only identified in the genomes of *Rugamonas* sp. A1-17 and *A. silviterrae* A2-55. Uptake systems for ferrous iron were also identified, the FeoAB uptake system was present in all Gram negative genomes and the EfeOB uptake system was present in the genome of *A. silviterrae* A2-55 (Table 3).

**TABLE 3:** Putative proteins related to iron transport identified in the bacterial strains genomes.

Classification	Conserved domains^b^	Protein ID^a^ in:				
		*Rugamonas* sp. A1-17	*Sphingomonas* sp. A2-49	*A. silviterrae* A2-55	*Rhodanobacter* sp. B2A1Ga-4^c^	*Undibacterium* sp. Jales W-56
Siderophore transport system	FepA: Outer membrane receptor for ferrienterochelin and colicins, COG4771 Fiu: Outer membrane receptor for monomeric catechols, COG4774 FhuE: Outer membrane receptor for ferric coprogen and ferric-rhodotorulic acid, COG4773 FecA: Outer membrane receptor for Fe^3+^ dicitrate, COG4772 Ferrichrome-iron receptor; Outer membrane receptor proteins, mostly Fe transport, COG1629	LPN04_01475 LPN04_04105 LPN04_15515 LPN04_15945 LPN04_18645 LPN04_18730 LPN04_20340 LPN04_24285 LPN04_27990 LPN04_16780 LPN04_24110 LPN04_27065 LPN04_20410 LPN04_25985 LPN04_17065 LPN04_17640 LPN04_26345	LPN01_15580 LPN01_06580 LPN01_07620 LPN01_14890 LPN01_08475		IMW82_05385 IMW82_00110	LPB67_14395 LPB67_07185 LPB67_04515

Iron ABC transporter type	FetB: ABC-type iron transport system FetAB, permease component,COG0390 FbpB: ABC-type Fe^3+^ transport system, permease component, COG1178 FepB: ABC-type Fe3+-hydroxamate transport system, periplasmic component, COG0614 FepC: ABC-type cobalamin/Fe3+-siderophores transport system, ATPase component, COG1120 FepD: ABC-type Fe3+-siderophore transport system, permease component, COG0609 FepG: ABC-type enterobactin transport system, permease component, COG4779 AfuA: ABC-type Fe^3+^ transport system, periplasmic component, COG1840	LPN04_31840 LPN04_20770 LPN04_20775	LPN01_08195 LPN01_08200	LPN03_11025 LPN03_12020 LPN03_11610 LPN03_11625 LPN03_11615 LPN03_11620 LPN03_12025	IMW82_11125	LPB67_10940 LPB67_13650 LPB67_04440 LPB67_04415 LPB67_04410 LPB67_10935

Ferrous iron transport system of the Feo or Efe type	Fe^2+^ transport system protein FeoA, COG1918 Fe^2+^ transport system protein B, COG0370 EfeB: Periplasmic deferrochelatase/peroxidase EfeB, COG2837 EfeO: Iron uptake system EfeUOB, periplasmic (or lipoprotein) component EfeO/EfeM, COG2822	LPN04_30530 LPN04_30525	LPN01_11090 LPN01_11095	LPN03_11695 LPN03_11700	IMW82_00670 MW82_00675	LPB67_06200 LPB67_06205

TonB-protein	Periplasmic protein TonB, links inner and outer membranes, COG0810	LPN04_01770 LPN04_02915 LPN04_18370 LPN04_18770 LPN04_18775 LPN04_20655 LPN04_23390 LPN04_30540 LPN04_32175 LPN04_31910	LPN01_05380 LPN01_01845		IMW82_07555 IMW82_08880 IMW82_17175	LPB67_06730 LPB67_01490 LPB67_14095 LPB67_14065 LPB67_16965 LPB67_17705

^a^Protein ID in NCBI Prokaryotic Genome Annotation Pipeline. ^b^Conserved domains obtained in NCBI database, COG (Clusters of Orthologous Groups) of proteins. ^c^Data from Caldeira et al. (2021).

### Minimum inhibitory concentration

The Minimum Inhibitory Concentration (MIC) for Ga and As(III) was determined in liquid assays in 96-multiwell plates. *Rhodanobacter* sp. B2A1Ga4 showed the highest MIC values for both metal(loid)s, 7.5 mM for As(III) and 2 mM for Ga, respectively. The lowest MIC values for As(III) were obtained with strains *A. silviterrae* A2-55 and *Undibacterium* sp. Jales W-56, while the strain *Sphingomonas* sp. A2-49 exhibited the lowest MIC value for Ga (Table 4).

**TABLE 4:** Minimum inhibitory concentration (MIC) for the different mine bacteria strains.

Bacteria strain	*Rugamonas*sp. A1-17	*Sphingomonas*sp. A2-49	*A. silviterrae*A2-55	*Rhodanobacter*sp.B2A1Ga4	*Undibacterium*sp.JalesW-56
**Gallium (III)**	1.5 mM	0.25 mM	2 mM	2 mM	1 mM
**Arsenite (III)**	3.75 mM	1 mM	0.5 mM	7.5 mM	0.5 mM

### Bioleaching experiments

#### Batch growth

**FIGURE 3:**
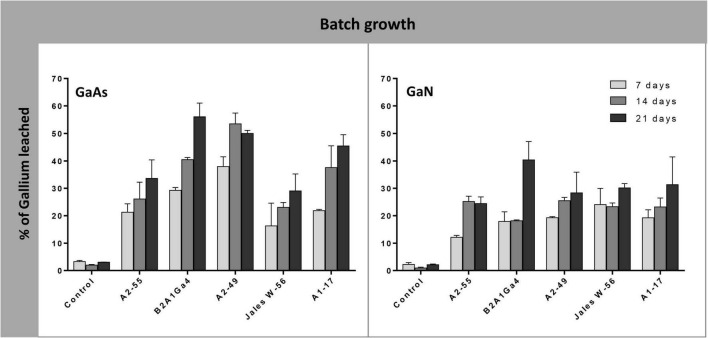
Gallium leaching from GaAs and GaN in batch growth of *Arthrobacter silviterrae* A2-55, *Rhodanobacter* sp. B2A1Ga4, *Sphingomonas* sp. A2-49, *Undibacterium* sp. Jales W-56, and *Rugamonas* sp. A1-17, with mR2Ab medium, pH = 6.0. Ga in leaching medium was determined at day 7, 14 and 21 of the experiments. Data are the mean values (± standard deviations) obtained from two or three independent experiments.

In batch growth assays where the bacterial strains were inoculated at the beginning of the bioleaching experiments, the efficiency of Ga leaching increased over time (factor “incubation time” was statistically relevant, *p* < 0.05) (Supplementary Data 4), and was maximum at 21 days, for most of the bacterial strains in both Ga mineral substrates, GaAs and GaN (Figure 3). The only exceptions were obtained with *Sphingomonas* sp. A2-49 and *A. silviterrae* A2-55 that reached the highest values of Ga leaching at 14 days of incubation from GaAs (53.0%) and from GaN (25.0%), respectively. Among the bacterial mine isolates, *Rhodanobacter* sp. B2A1Ga4 showed the highest efficiency to leach Ga from GaAs (56.0%) and from GaN (40%) (Figure 3). Together with *Sphingomonas* sp. A2-49 (53.0% leaching), for GaAs, the high leaching capacities were statistically significant when compared to the lowest performing strains: *A. silviterrae* A2-55 and *Undibacterium* sp. Jales W-56, which reached, respectively, only 33.6 and 29.0% of Ga leaching. Effectively, the later strains did not exhibit significant differences comparing leaching on days 7 and 21, unlike the other strains. All bacterial strains performed better in presence of GaAs (29.0 to 56.0%) than in GaN (24.4 to 40.4%) except *Undibacterium* sp. Jales W-56, with statistical significance (*p* < 0.05) after 21 days of experiment (Figure 3).

#### Cultures in different phases of growth

**FIGURE 4:**
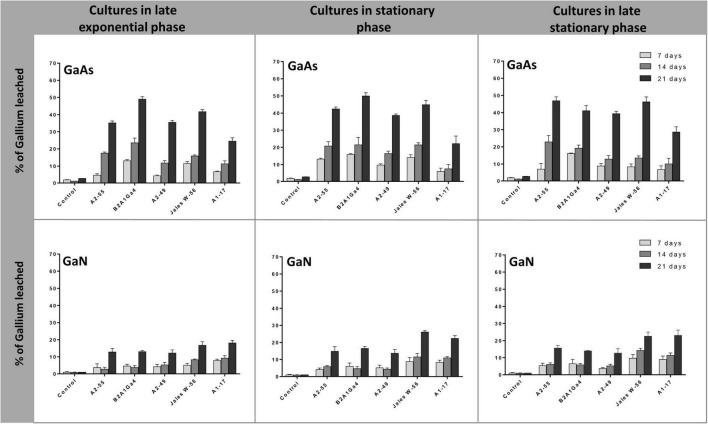
Gallium leaching from GaAs and GaN by bacterial cultures at different growth phases (late exponential, 8 h; stationary, 24 h, and late stationary, 48 h). Strains: *Arthrobacter silviterrae* A2-55, *Rhodanobacter* sp. B2A1Ga4, *Sphingomonas* sp. A2-49, *Undibacterium* sp. Jales W-56, and *Rugamonas* sp. A1-17. Ga in leaching medium was determined at day 7, 14, and 21 of the experiments. Data are the mean values (± standard deviations) obtained from two or three independent experiments.

In these bioleaching experiments, bacterial strains were first grown under their optimum conditions until they reached the desired phase of growth (late exponential, 8 h; stationary, 24 h, and late stationary, 48 h (Supplementary Data 4). The cultures at different phases of growth were then used as leaching media in the assays. For all bacteria strains, the efficiency of Ga leaching of cultures at the three different phases of growth was similar for GaAs, with marginal differences of 1.0 to 12.0% in Ga leaching capacity between them, and no statistical significance (Figure 4). This trend was observed at all incubation times (7, 14, and 21 days). The factor “growth phase” was statistically relevant when analyzing the results obtained with GaN. The use of cultures, in the stationary phase or in late stationary phase, significantly increased the Ga leaching by 5.0–9.0% after 21 days, for both strains *Undibacterium* sp. Jales W-56 and *Rugamonas* sp. A1-17 comparatively to the Ga leaching using cultures in the late exponential phase (Figure 4). The results obtained with GaN separated these two strains, with a higher leaching ability (*p* < 0.05), from strains *A. silviterrae* A2-55, *Rhodanobacter* sp. B2A1Ga4, and *Sphingomonas* sp. A2-49 with a lower leaching capacity on days 14 and 21. Significantly higher efficiency of Ga leaching was always obtained at 21 days for both GaN and GaAs.

#### Cell-free spent medium

**FIGURE 5:**
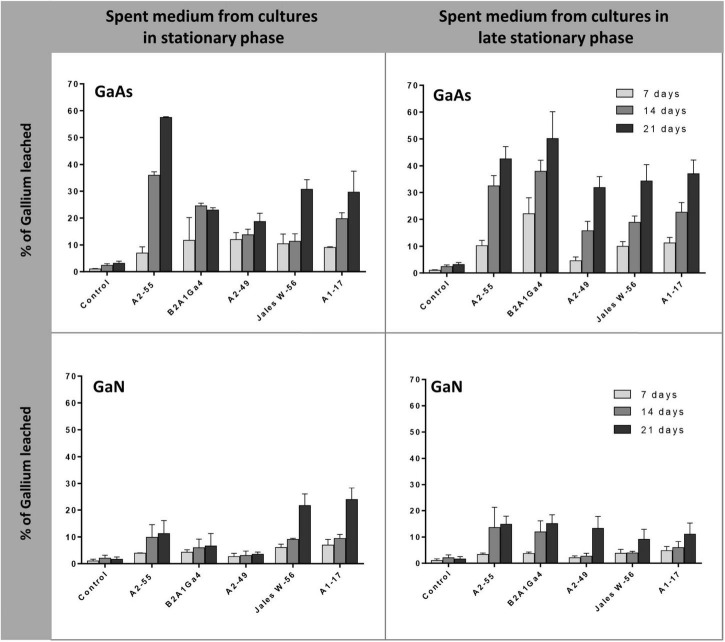
Gallium leaching from GaAs and GaN by cell-free spent medium of cultures at different phases (stationary, 24 h, and late stationary, 48 h). Strains: *Arthrobacter silviterrae* A2-55, *Rhodanobacter* sp. B2A1Ga4, *Sphingomonas* sp. A2-49, *Undibacterium* sp. Jales W-56, and *Rugamonas* sp. A1-17. Ga in leaching medium was determined at day 7, 14, and 21 of the experiments. Data are the mean values (± standard deviations) obtained from two or three independent experiments.

Cell-free spent medium from cultures at stationary phase of growth (24 h) and late stationary phase of growth (48 h) was also used to evaluate the efficiency of Ga leaching from GaAs and GaN. The results showed that spent medium from *A. silviterrae* A2-55 at stationary phase had the highest efficiency to leach Ga from GaAs, reaching 35.9 and 57.0% after 14 and 21 days, respectively, significantly outperforming all other strains (Figure 5). However, the use of the spent medium at late stationary phase from all the other 4 strains improved their Ga leaching performance from GaAs. This enhancement was clearly evident (*p* < 0.05) for *Rhodanobacter* sp. B2A1Ga4, that showed an increase of soluble Ga amounts of 13.0 and 27.0% using the cell-free spent medium from cultures at late stationary growth phase, after 14 and 21 days, respectively. Cell free-spent medium from all strains showed low efficiency of Ga leaching from GaN. The maximum Ga leaching amounts from GaN, 21.7 and 23.9%, were obtained using cell free-spent medium from cultures of *Undibacterium* sp. Jales W-56 and *Rugamonas* sp. A1-17 at stationary phase, respectively, after 21 days. Both strains performance differed significantly from the other three strains. However, the use of medium of late stationary phase improved significantly the Ga leaching efficiency of the three other strains, particularly on day 21.

#### pH variation

pH values were measured in the beginning and during the bioleaching experiments after 7, 14, and 21 days. pH changes were observed in all the different bioleaching experiments used in this study. In batch experiments the initial pH of mR2Ab was adjusted to 6.0 and by day 7 the pH value was between 7.0 and 7.8, and at the end of the experiments, the pH values reached 8.0 or 8.5. In the bioleaching experiments with cultures or spent medium in different phases of growth as leaching medium, the initial pH was higher between 6.8 and 7.9, since the bacterial strains have previously grown in this medium. pH changes throughout the assays were also observed, and after 21 days the pH reached values of between 8.0 and 9.0. However, the pH values of the inoculated controls did not have major changes and remained between 6.0 and 6.5 throughout the assays.

## Discussion

Bacteria that inhabit metal-contaminated environments, such as mines, play unique roles in the biochemical cycling of metals, including mineral dissolution by leaching processes and metal recovery. The present study combines experimental and whole-genome analysis approaches to investigate the ability of five distinct bacterial mine isolates to leach and mobilize Ga from GaAs and GaN.

All these bacterial genomes shared a large number of homologous genes that were assigned to COG functional categories, of which the “amino acid transport and metabolism” [E], “translation, ribosomal structure and biogenesis” [J], and “replication, recombination, and repair” [L] were the most abundant. These findings show that these heterotrophic bacteria have the ability to synthesize amino acids as energy supply [E], and that the enrichment in genes coding for structures involved in protein synthesis [J] and DNA repair [L] might provide an adaptive strategy to cope with high metal concentrations of their natural environment. High concentrations of heavy metals can cause damage to bacterial cells (Chandrangsu et al., 2017; Igiri et al., 2018) and mechanisms of defense such as the synthesis of proteins involved in transport and detoxification, and also proteins involved in DNA repair can be trigged as rescue systems (Zhang et al., 2018; Mathivanan et al., 2021).

Genome analysis of these five strains showed predicted genes for the biosynthetic pathways of most amino acids, indicating that they potentially can synthesize most of the amino acids. The presence of these amino acids in the growth supernatant of cultures of these strains could have an important role in the Ga leaching mechanism (Supplementary Data 4). Previous studies have already pointed out that amino acids/peptides/proteins produced by heterotrophic bacteria were involved in Ga leaching from GaAs and GaN (Maneesuwannarat et al., 2016a,b). More recently, Kudpeng et al. (2021) reported that amino acids/peptides/proteins produced by *Macrococcus caseolyticus* and *Acinetobacter calcoaceticus* were capable of gold bioleaching from silicate ore. The Ga leaching ability of these molecules is related to the presence of charges in their structure, which varies with different pHs due to the pK values of the carboxylic and amino groups. It was shown that basic pH conditions promoted deprotonation of the carboxylic and amino groups of amino acids/peptides/proteins, resulting in a higher number of negative charges and interaction with positively charged Ga (Maneesuwannarat et al., 2016a,b, 2019). In the bioleaching experiments of this study, the progressive increase of Ga in the leachate medium was always accompanied by an increase of pH values, reaching values between 8 and 9, after 21 days of incubation. This pH range also seemed to favor the bioleaching process, as the highest values of Ga leaching were obtained at 21 days in most bioleaching assays. These alkaline pH values also showed that these bacterial strains do not produce organic acids as a strategy to mobilize Ga. The comparison of the different bioleaching assays demonstrated a divergence in bioleaching efficiency among the bacterial strains, which could be correlated with the different metabolic features presented in their genomes. Thus, the bacterial genomes were analyzed to unveil the mechanisms of resistance to arsenic (As) and Ga, the two metal(loid)s used in the bioleaching assays. All bacterial genomes harbored several clusters of *ars* genes, with a different variety and combination of genes involved in the As resistance. Strain *Rhodanobacter* sp. B2A1Ga4, with the highest As resistance, exhibited multiple and redundant *ars* genes in complex *ars* gene clusters in its genome in comparison to all other genomes. Furthermore, the presence of an *arsA* gene within the *arsRCDAB* cluster enhances the efflux activity of ArsB, by coupling the ATP-hydrolyzing activity of an ATPase, increasing As (III) extrusion and resistance to this metalloid (Firrincieli et al., 2019). Strains *Rugamonas* sp. A1-17 and *Sphingomonas* sp. A2-49 with MICs values of 3.75 and 1 mM, respectively, harbored *arsRCBH* gene clusters. The presence of an additional *arsN2* gene in the *ars* gene clusters of *Sphingomonas* sp. A2-49 was an interesting finding. This gene is often found in *ars* operons, suggesting a role of the ArsN proteins in arsenic resistance. However, the exact functions of ArsN proteins are not yet known (Chauhan et al., 2009; Chen et al., 2016; Ben-Fekih et al., 2018). Strains *A. silviterrae* A2-55 and *Undibacterium* sp. Jales W-56 exhibited low As resistance (MIC = 0.5 mM). Genomic evidences showed simpler *ars* gene clusters with variations of the canonical *arsRBC* cluster, where the *arsB* gene was replaced by *acr3* or aquaporin Z gene. Although the role of ACR3 protein as an efflux pump is well known (Fu et al., 2009; Ben-Fekih et al., 2018), the role of aquaporins in As resistance is more recent. The aquaporin Z genes identified in these genomes encode for aquaglyceroporins GlpF, which usually transport water and organic solutes such as glycerol (Borgnia et al., 1999), but can also function as an As (III) efflux pump replacing the ArsB transporter (Yang et al., 2012; Mukhopadhyay et al., 2014; Yang et al., 2015). Literature reports strains, such as *Ochrobactrum tritici* (Branco et al., 2008; Sousa et al., 2015) or *Herminiimonas arsenicoxydans* (Muller et al., 2006), as having a high number of *ars* genes, which confer high resistance to arsenic. Multiple and redundant *ars* genes in Prokaryotes commonly give rise to higher levels of resistance to arsenic (Li and Krumholz, 2007). Additionally, these redundant *ars* genes may be expressed differentially depending on the environmental conditions, which may constitute an advantage when facing arsenic stress (Ben-Fekih et al., 2018).

Currently, little information is available on the mechanisms of Ga resistance. Nonetheless, studies indicate that iron metabolism plays an important role in Ga resistance and cellular protection against the potential toxicity of this metal (Gugala et al., 2019; Caldeira et al., 2021). It is predominantly assumed that Ga crosses cell membranes using the iron transport systems, in particular the Fe-siderophore transport system, as Ga is considered an iron mimetic (Gugala et al., 2019; Li et al., 2022). Among the bacterial strains tested in the current study, the highest Ga resistance was demonstrated by *Rhodanobacter* sp. B2A1Ga4 and *A. silviterrae* A2-55 (MIC = 2 mM). A lower number of genes encoding iron transporter systems were identified in *Rhodanobacter* sp. B2A1Ga4 genome in comparison to other genomes, might prevent Ga import and accumulation, resulting in higher Ga resistance. A noteworthy finding was the presence of a gene encoding for a putative FetB protein, in the genome of *A. silviterrae* A2-55, which is reported as an iron efflux system (Nicolaou et al., 2013). This system can be used by these cells to extrude Ga, increasing the Ga resistance. Strains *Rugamonas* sp. A1-17 and *Undibacterium* sp. Jales W-56 showed an intermediate Ga resistance (MICs = 1.5–1.0 mM), and a high number of proteins involved in iron transport were predicted in these genomes. However, in the genome of *Rugamonas* sp. A1-17 is also predicted a putative FetB protein, as in genome of *A. silviterrae* A2-55, which may contribute to the Ga resistance of this strain. The lowest resistance to Ga was presented by *Sphingomonas* sp. A2-49 (MIC 0.25 mM) which carries a large number of putative outer membrane receptors for Fe-siderophores complexes, providing a high number of potential Ga targets. Ga resistance related to iron transport systems, has also been reported in other microorganisms. Ga resistance in *P. aeruginosa* increased after the inactivation of the *hitA* gene encoding an iron transporter (García-Contreras et al., 2013). In *E. coli*, the deletion of genes encoding proteins involved in Fe-siderophores import complexes, such as the FepG, FecA and the accessory TonB proteins, reduced Ga import and intracellular accumulation of this metal (Graves et al., 2019; Gugala et al., 2019). Additionally, a previous study with a mutant of *Rhodanobacter* sp. B2A1Ga4, in which the ferrous iron FeoAB uptake system was inactivated, showed that acquisition of iron by this system is critical to control the oxidative stress in presence of indium and Ga, enhancing the resistance to both metals (Caldeira et al., 2021).

The presence of effective resistance mechanisms to Ga and As was particularly important in GaAs bioleaching batch assays where bacterial growth occur simultaneously with the mobilization of Ga and As into the leaching medium. Actually, under this condition, a correlation could be established: strains with higher resistance, mainly to As, performed better in this bioleaching assays, such as *Rhodanobacter* sp. B2A1Ga4 and *Sphingomonas* sp. A2-49. On the other hand, strains with lower resistance, such as *A. silviterrae* A2-55 and *Undibacterium* sp. Jales W-56, were not so efficient in the bioleaching process. However, in GaAs bioleaching assays, where cultures were first grown in absence of GaAs and then used either as it is or as cell-free spent medium, both strains clearly improved their bioleaching efficiency. In fact, *A. silviterrae* A2-55 outperformed all the other stains (57.0% Ga leached) with cell-free spent medium from the stationary phase of growth.

In this work, the genes involved in nitrogen metabolism were not deeply explored, since these heterotrophic bacteria naturally have mechanisms to deal with the nitrogen released from the bioleaching assays with GaN. In Ga bioleaching assays from GaN, the presence of active bacterial cells seems to be important, since free-cell spent medium assays presented the lowest values of Ga leaching (2.0 to 24.0%). Moreover, older cultures favored the bioleaching process from GaN, indicating that the metabolic by-products produced in the late stages of growth and the pH provided more efficient conditions for Ga mobilization. Overall, the efficiency of Ga bioleaching from GaN was lower when compared to GaAs. The refractory property of GaN generated difficulties in the process of bioleaching, and it was shown that pretreatment with high temperatures improved the efficiency of the bioleaching process (Maneesuwannarat et al., 2016b).

The integrative approach that combines the experimental and genomic analysis undertaken in this work showed that the genetic features related to amino acids metabolism and genetic mechanisms that potentially account for Ga and As resistance in these bacterial mine strains are related to their different ability to mobilize and leach Ga from GaAs and GaN. Genomic analyses to identify metabolic traits linked to the bioleaching process as presented here improve our current knowledge and further promote the industrial applications of bioleaching technologies.

## Data availability statement

The datasets presented in this study can be found in online repositories. The names of the repository/repositories and accession number(s) can be found below: https://www.ncbi.nlm.nih.gov/genbank/, for JAJLPB000000000 (*Rugamonas* sp. A1-17); for JAJLPA000000000 (*Sphingomonas* sp. A2-49); for JAJLOZ000000000 (Arthrobacter silviterrae A2-55); for JAJLQW000000000 (*Undibacterium* sp. Jales W-56); and for JADBJR000000000 (*Rhodanobacter* sp. B2A1Ga4).

## Author contributions

AC and RB conceived and designed the experiments and wrote the manuscript. AC performed the genome analysis and the experiments. AC, RF, PM, and RB analyzed the data. RF performed the statistical analysis. RB and PM contributed with reagents, materials, and analysis tools. All authors revised and approved the manuscript.

## Footnotes

1
http://jspecies.ribohost.com/jspeciesws

2
http://bioinformatics.psb.ugent.be/webtools/Venn/

3
www.graphpad.com
