# Supplementary material for: Genome mining to unravel potential metabolic pathways linked to gallium bioleaching ability of bacterial mine isolates
Source: Front Microbiol. 2022 Sep 13;13:970147. doi: 10.3389/fmicb.2022.970147 (PMC9518604; doi:10.3389/fmicb.2022.970147)
Supplement: Supplementary file 1 [file Data_Sheet_1.PDF]

TYPE (STRAIN) GENOME SERVER

JOB SUMMARY

PRINT DATE: 2022-01-12 12:43:31 +0100

JOB ID: 0b91f331-43d5-477d-b081-42ba02a327fb

RESULT PAGE: https://tygs.dsmz.de/user_results/show?guid=0b91f331-43d5-477d-b081-42ba02a327fb

Table 1: Phylogenies

Publication-ready versions of both the genome-scale GBDP tree and the 16S rRNA gene sequence tree can be customized and exported either in SVG (vector graphic) or PNG format from within the phylogeny viewers in your TYGS result page. For publications the **SVG format is recommended** because it is lossless, always keeps its high resolution and can also be easily converted to other popular formats such as PDF or EPS. Please follow the link provided above!

Table 2: Identification

The below list contains the result of the TYGS species identification routine.

Explanation of remarks that might occur in the below table:

remark [R1]: The TYGS type strain database is automatically updated on an almost daily basis. However, if a particular type strain genome is not available in the TYGS database, this can have several reasons which are detailed in the FAQ. You can request an extended 16S rRNA gene analysis via the 16S tree viewer found in your result page to detect **not yet genome-sequenced** type strains relevant for your study.

remark [R2]: > 70% dDDH value (formula d_4) and (almost) minimal dDDH values for gene-content formulae d_0 and d_6 indicate a potentially unreliable identification result and should thus be checked via the 16S rRNA gene sequence similarity. Such strong deviations can, in principle, be caused by sequence contamination.

remark [R3]: G+C content difference of > 1 % indicates a potentially unreliable identification result because within species G+C content varies no more than 1 %, if computed from genome sequences (PMID: 24505073).

Strain	Conclusion	Identification result	Remark
**'A2-55.fna'**	belongs to known species	*Arthrobacter silviterrae*	

Table 3: Pairwise comparisons of user genomes vs. type-strain genomes

The following table contains the pairwise dDDH values between your user genomes and the selected type-strain genomes. The dDDH values are provided along with their confidence intervals (C.I.) for the three different GBDP formulas:

- formula d_0 (a.k.a. GGDC formula 1): length of all HSPs divided by total genome length
- formula d_4 (a.k.a. GGDC formula 2): sum of all identities found in HSPs divided by overall HSP length
- formula d_6 (a.k.a. GGDC formula 3): sum of all identities found in HSPs divided by total genome length

Note: Formula d_4 is independent of genome length and is thus robust against the use of incomplete draft genomes. For other reasons for preferring formula d_4, see the FAQ.

Query	Subject	d_0	C.I. d_0	d_4	C.I. d_4	d_6	C.I. d_6	Diff. G+C Percent
**'A2-55.fna.fna'**	*Arthrobacter silviterrae* DSM 27180	80.6	[76.7 - 84.0]	83.1	[80.3 - 85.6]	84.0	[80.7 - 86.8]	0.07
**'A2-55.fna.fna'**	*Arthrobacter dokdonellae* DCT-5	30.4	[27.1 - 34.0]	24.5	[22.2 - 27.0]	28.0	[25.1 - 31.1]	0.55
**'A2-55.fna.fna'**	*Arthrobacter livingstonensis* LI2	27.5	[24.2 - 31.2]	23.9	[21.6 - 26.4]	25.7	[22.8 - 28.8]	0.89
**'A2-55.fna.fna'**	*Arthrobacter stackebrandtii* DSM 16005	22.4	[19.1 - 26.0]	22.5	[20.2 - 25.0]	21.4	[18.6 - 24.5]	0.13
**'A2-55.fna.fna'**	*Arthrobacter alpinus* DSM 22274	17.9	[14.9 - 21.5]	21.3	[19.0 - 23.7]	17.7	[15.0 - 20.6]	5.1
**'A2-55.fna.fna'**	*Arthrobacter glacialis* CGMCC 1.10025	19.3	[16.1 - 22.9]	21.2	[18.9 - 23.6]	18.8	[16.1 - 21.8]	4.28
**'A2-55.fna.fna'**	*Specibacter cremeus* C1-50	18.7	[15.6 - 22.3]	21.1	[18.9 - 23.5]	18.3	[15.7 - 21.3]	3.13
**'A2-55.fna.fna'**	*Arthrobacter ipsi* IA7	14.5	[11.7 - 17.9]	20.8	[18.6 - 23.2]	14.7	[12.3 - 17.6]	0.35
**'A2-55.fna.fna'**	*Arthrobacter psychrochitiniphilus* DSM 23143	17.2	[14.2 - 20.7]	20.6	[18.4 - 23.1]	17.0	[14.4 - 19.9]	6.03
**'A2-55.fna.fna'**	*Arthrobacter psychrolactophilus* B7	17.1	[14.1 - 20.6]	20.6	[18.4 - 23.0]	16.9	[14.3 - 19.9]	5.02
**'A2-55.fna.fna'**	*Pseudarthrobacter enclensis* NIO-1008	15.0	[12.1 - 18.4]	20.3	[18.1 - 22.7]	15.1	[12.6 - 18.0]	1.4
**'A2-55.fna.fna'**	*Arthrobacter liuii* CGMCC1.12778	14.7	[11.9 - 18.1]	20.2	[18.0 - 22.6]	14.9	[12.4 - 17.7]	0.23
**'A2-55.fna.fna'**	*Arthrobacter cryoconiti* Cr6-08	17.1	[14.0 - 20.6]	19.8	[17.6 - 22.2]	16.8	[14.2 - 19.8]	7.2

TYPE (STRAIN) GENOME SERVER

JOB SUMMARY

PRINT DATE:	2022-01-12 11:42:08 +0100
JOB ID:	fca03686-cb67-4f36-b4f2-64696f1295ac
RESULT PAGE:	https://tygs.dsmz.de/user_results/show?guid=fca03686-cb67-4f36-b4f2-64696f1295ac

Table 1: Phylogenies

Publication-ready versions of both the genome-scale GBDP tree and the 16S rRNA gene sequence tree can be customized and exported either in SVG (vector graphic) or PNG format from within the phylogeny viewers in your TYGS result page. For publications the **SVG format is recommended** because it is lossless, always keeps its high resolution and can also be easily converted to other popular formats such as PDF or EPS. Please follow the link provided above!

Table 2: Identification

The below list contains the result of the TYGS species identification routine.

Explanation of remarks that might occur in the below table:

remark [R1]: The TYGS type strain database is automatically updated on an almost daily basis. However, if a particular type strain genome is not available in the TYGS database, this can have several reasons which are detailed in the FAQ. You can request an extended 16S rRNA gene analysis via the 16S tree viewer found in your result page to detect **not yet genome-sequenced** type strains relevant for your study.

remark [R2]: > 70% dDDH value (formula d_4) and (almost) minimal dDDH values for gene-content formulae d_0 and d_6 indicate a potentially unreliable identification result and should thus be checked via the 16S rRNA gene sequence similarity. Such strong deviations can, in principle, be caused by sequence contamination.

remark [R3]: G+C content difference of > 1 % indicates a potentially unreliable identification result because within species G+C content varies no more than 1 %, if computed from genome sequences (PMID: 24505073).

Strain	Conclusion	Identification result	Remark
**'A1-17'**	potential new species		see [R1]

Table 3: Pairwise comparisons of user genomes vs. type-strain genomes

The following table contains the pairwise dDDH values between your user genomes and the selected type-strain genomes. The dDDH values are provided along with their confidence intervals (C.I.) for the three different GBDP formulas:

• formula d_0 (a.k.a. GGDC formula 1): length of all HSPs divided by total genome length
• formula d_4 (a.k.a. GGDC formula 2): sum of all identities found in HSPs divided by overall HSP length
• formula d_6 (a.k.a. GGDC formula 3): sum of all identities found in HSPs divided by total genome length

Note: Formula d_4 is independent of genome length and is thus robust against the use of incomplete draft genomes. For other reasons for preferring formula d_4, see the FAQ.

Query	Subject	d_0	C.I. d_0	d_4	C.I. d_4	d_6	C.I. d_6	Diff. G+C Percent
**'A1-17.fna'**	*Rugamonas rivuli* FT103W	57.2	[53.6 - 60.7]	38.1	[35.6 - 40.6]	52.9	[49.7 - 55.9]	0.48
**'A1-17.fna'**	*Rugamonas aquatica* FT29W	53.9	[50.4 - 57.4]	37.8	[35.3 - 40.3]	50.2	[47.1 - 53.2]	0.61
**'A1-17.fna'**	*Rugamonas aceris* SAP-35	38.5	[35.1 - 42.0]	29.2	[26.8 - 31.7]	35.4	[32.4 - 38.4]	0.44
**'A1-17.fna'**	*Duganella aquatilis* FT26W	37.4	[34.1 - 40.9]	26.9	[24.6 - 29.4]	33.9	[30.9 - 37.0]	0.64
**'A1-17.fna'**	*Duganella alba* FT9W	38.6	[35.2 - 42.0]	26.7	[24.4 - 29.2]	34.6	[31.7 - 37.7]	0.42
**'A1-17.fna'**	*Duganella callida* DN04T	34.5	[31.1 - 38.0]	26.0	[23.7 - 28.5]	31.4	[28.5 - 34.5]	0.02
**'A1-17.fna'**	*Duganella radicis* KCTC22382	37.8	[34.5 - 41.3]	25.7	[23.4 - 28.2]	33.8	[30.8 - 36.9]	0.86
**'A1-17.fna'**	*Duganella margarita* FT109W	36.6	[33.2 - 40.1]	25.7	[23.4 - 28.2]	32.9	[29.9 - 36.0]	1.21
**'A1-17.fna'**	*Duganella lactea* FT50W	34.6	[31.3 - 38.2]	25.6	[23.3 - 28.1]	31.4	[28.5 - 34.5]	1.14
**'A1-17.fna'**	*Rugamonas rubra* ATCC 43154	28.5	[25.1 - 32.1]	25.2	[22.9 - 27.7]	26.7	[23.8 - 29.8]	2.58
**'A1-17.fna'**	*Duganella guangzhouensis* FT80W	32.7	[29.3 - 36.3]	25.1	[22.8 - 27.6]	29.9	[26.9 - 33.0]	1.97
**'A1-17.fna'**	*Duganella rivi* FT55W	34.8	[31.4 - 38.3]	25.0	[22.7 - 27.5]	31.3	[28.4 - 34.4]	2.67
**'A1-17.fna'**	*Duganella fentianensis* FT93W	28.5	[25.1 - 32.1]	23.8	[21.5 - 26.3]	26.4	[23.5 - 29.5]	3.47
**'A1-17.fna'**	*Janthinobacterium psychrotolerans* S3-2	20.4	[17.2 - 24.0]	22.0	[19.7 - 24.4]	19.8	[17.0 - 22.8]	1.36
**'A1-17.fna'**	*Massilia arenosa* MC02	19.0	[15.8 - 22.5]	20.8	[18.6 - 23.3]	18.5	[15.8 - 21.5]	1.75
**'A1-17.fna'**	*Massilia ginsengisoli* KCTC 42409	17.5	[14.4 - 21.0]	20.7	[18.4 - 23.1]	17.2	[14.6 - 20.2]	3.57

PRINT DATE:	2022-01-12 15:01:14 +0100
JOB ID:	3e2adc32-53ad-44ae-a941-e2bd7dafa66e
RESULT PAGE:	https://tygs.dsmz.de/user_results/show?guid=3e2adc32-53ad-44ae-a941-e2bd7dafa66e

Table 1: Phylogenies

Publication-ready versions of both the genome-scale GBDP tree and the 16S rRNA gene sequence tree can be customized and exported either in SVG (vector graphic) or PNG format from within the phylogeny viewers in your TYGS result page. For publications the **SVG format is recommended** because it is lossless, always keeps its high resolution and can also be easily converted to other popular formats such as PDF or EPS. Please follow the link provided above!

Table 2: Identification

The below list contains the result of the TYGS species identification routine.

Explanation of remarks that might occur in the below table:

remark [R1]: The TYGS type strain database is automatically updated on an almost daily basis. However, if a particular type strain genome is not available in the TYGS database, this can have several reasons which are detailed in the FAQ. You can request an extended 16S rRNA gene analysis via the 16S tree viewer found in your result page to detect **not yet genome-sequenced** type strains relevant for your study.

remark [R2]: > 70% dDDH value (formula d_4) and (almost) minimal dDDH values for gene-content formulae d_0 and d_6 indicate a potentially unreliable identification result and should thus be checked via the 16S rRNA gene sequence similarity. Such strong deviations can, in principle, be caused by sequence contamination.

remark [R3]: G+C content difference of > 1 % indicates a potentially unreliable identification result because within species G+C content varies no more than 1 %, if computed from genome sequences (PMID: 24505073).

Strain	Conclusion	Identification result	Remark
**'A2-49FNA'**	potential new species		see [R1]

Table 3: Pairwise comparisons of user genomes vs. type-strain genomes

The following table contains the pairwise dDDH values between your user genomes and the selected type-strain genomes. The dDDH values are provided along with their confidence intervals (C.I.) for the three different GBDP formulas:

• formula d_0 (a.k.a. GGDC formula 1): length of all HSPs divided by total genome length
• formula d_4 (a.k.a. GGDC formula 2): sum of all identities found in HSPs divided by overall HSP length
• formula d_6 (a.k.a. GGDC formula 3): sum of all identities found in HSPs divided by total genome length

Note: Formula d_4 is independent of genome length and is thus robust against the use of incomplete draft genomes. For other reasons for preferring formula d_4, see the FAQ.

Query	Subject	d_0	C.I. d_0	d_4	C.I. d_4	d_6	C.I. d_6	Diff. G+C Percent
**'A2-49FNA.fna'**	*Sphingomonas insulae* DSM 21792	40.1	[36.7 - 43.5]	27.0	[24.6 - 29.5]	35.8	[32.9 - 38.9]	1.69
**'A2-49FNA.fna'**	*Sphingomonas aquatilis* DSM 15581	34.0	[30.6 - 37.5]	25.2	[22.8 - 27.6]	30.8	[27.9 - 33.9]	1.31
**'A2-49FNA.fna'**	*Sphingomonas melonis* DAPP-PG 224	33.0	[29.6 - 36.6]	25.1	[22.8 - 27.6]	30.1	[27.1 - 33.2]	1.41
**'A2-49FNA.fna'**	*Sphingomonas rubra* CGMCC 1.9113	22.7	[19.5 - 26.4]	21.6	[19.4 - 24.1]	21.6	[18.8 - 24.6]	0.38
**'A2-49FNA.fna'**	*Sphingomonas abaci* DSM 15867	21.4	[18.2 - 25.1]	21.2	[19.0 - 23.6]	20.5	[17.7 - 23.5]	0.5
**'A2-49FNA.fna'**	*Sphingomonas ginsenosidimutans* KACC 14949	20.9	[17.7 - 24.5]	20.9	[18.7 - 23.3]	20.0	[17.3 - 23.1]	0.37
**'A2-49FNA.fna'**	*Sphingomonas aeria* B093034T	21.7	[18.4 - 25.3]	20.8	[18.6 - 23.2]	20.6	[17.9 - 23.7]	1.33
**'A2-49FNA.fna'**	*Sphingomonas carotinifaciens* DSM 27347	21.4	[18.1 - 25.0]	20.7	[18.4 - 23.1]	20.4	[17.6 - 23.4]	1.39
**'A2-49FNA.fna'**	*Sphingomonas hominis* HHU CXW	19.2	[16.1 - 22.8]	20.6	[18.4 - 23.0]	18.7	[16.0 - 21.7]	1.26
**'A2-49FNA.fna'**	*Sphingomonas palmae* JS21-1T	19.9	[16.7 - 23.5]	20.3	[18.1 - 22.7]	19.2	[16.5 - 22.2]	1.06
**'A2-49FNA.fna'**	*Sphingomonas pruni* NBRC 15498	16.2	[13.3 - 19.7]	20.0	[17.8 - 22.4]	16.1	[13.6 - 19.1]	3.59
**'A2-49FNA.fna'**	*Sphingomonas panacisoli* HKS19	17.2	[14.1 - 20.7]	19.9	[17.7 - 22.3]	16.9	[14.3 - 19.9]	3.26
**'A2-49FNA.fna'**	*Sphingomonas asaccharolytica* NBRC 15499	16.2	[13.2 - 19.6]	19.8	[17.6 - 22.2]	16.1	[13.5 - 19.0]	3.77
**'A2-49FNA.fna'**	*Sphingomonas leidyi* DSM 4733	16.5	[13.5 - 20.0]	19.8	[17.6 - 22.2]	16.4	[13.8 - 19.3]	0.83
**'A2-49FNA.fna'**	*Sphingomonas mali* NBRC 15500	16.2	[13.2 - 19.7]	19.8	[17.6 - 22.2]	16.1	[13.6 - 19.0]	3.55

TYPE (STRAIN) GENOME SERVER

JOB SUMMARY

PRINT DATE:	2022-01-12 14:59:49 +0100
JOB ID:	5b3295c5-04c9-4e93-8991-22affe1b4243
RESULT PAGE:	https://tygs.dsmz.de/user_results/show?guid=5b3295c5-04c9-4e93-8991-22affe1b4243

Table 1: Phylogenies

Publication-ready versions of both the genome-scale GBDP tree and the 16S rRNA gene sequence tree can be customized and exported either in SVG (vector graphic) or PNG format from within the phylogeny viewers in your TYGS result page. For publications the **SVG format is recommended** because it is lossless, always keeps its high resolution and can also be easily converted to other popular formats such as PDF or EPS. Please follow the link provided above!

Table 2: Identification

The below list contains the result of the TYGS species identification routine.

Explanation of remarks that might occur in the below table:

remark [R1]: The TYGS type strain database is automatically updated on an almost daily basis. However, if a particular type strain genome is not available in the TYGS database, this can have several reasons which are detailed in the FAQ. You can request an extended 16S rRNA gene analysis via the 16S tree viewer found in your result page to detect **not yet genome-sequenced** type strains relevant for your study.

remark [R2]: > 70% dDDH value (formula d_4) and (almost) minimal dDDH values for gene-content formulae d_0 and d_6 indicate a potentially unreliable identification result and should thus be checked via the 16S rRNA gene sequence similarity. Such strong deviations can, in principle, be caused by sequence contamination.

remark [R3]: G+C content difference of > 1 % indicates a potentially unreliable identification result because within species G+C content varies no more than 1 %, if computed from genome sequences (PMID: 24505073).

Strain	Conclusion	Identification result	Remark
**'Ga-4'**	potential new species		see [R1]

Table 3: Pairwise comparisons of user genomes vs. type-strain genomes

The following table contains the pairwise dDDH values between your user genomes and the selected type-strain genomes. The dDDH values are provided along with their confidence intervals (C.I.) for the three different GBDP formulas:

- formula d_0 (a.k.a. GGDC formula 1): length of all HSPs divided by total genome length
- formula d_4 (a.k.a. GGDC formula 2): sum of all identities found in HSPs divided by overall HSP length
- formula d_6 (a.k.a. GGDC formula 3): sum of all identities found in HSPs divided by total genome length

Note: Formula d_4 is independent of genome length and is thus robust against the use of incomplete draft genomes. For other reasons for preferring formula d_4, see the FAQ.

Query	Subject	d_0	C.I. d_0	d_4	C.I. d_4	d_6	C.I. d_6	Diff. G+C Percent
**'Ga-4.fna'**	*Rhodanobacter denitrificans* 2APBS1	56.1	[52.5 - 59.6]	39.9	[37.4 - 42.4]	52.6	[49.5 - 55.7]	0.78
**'Ga-4.fna'**	*Rhodanobacter thiooxydans* LCS2	52.6	[49.1 - 56.1]	37.9	[35.5 - 40.5]	49.2	[46.1 - 52.2]	0.52
**'Ga-4.fna'**	*Rhodanobacter spathiphylli* B39	43.1	[39.8 - 46.6]	29.7	[27.3 - 32.2]	39.0	[36.0 - 42.1]	0.16
**'Ga-4.fna'**	*Rhodanobacter panaciterrae* KCTC 22232	42.4	[39.0 - 45.8]	27.1	[24.8 - 29.6]	37.5	[34.6 - 40.6]	3.01
**'Ga-4.fna'**	*Rhodanobacter fulvus* Jip2	30.8	[27.4 - 34.4]	25.3	[22.9 - 27.7]	28.5	[25.5 - 31.6]	1.08
**'Ga-4.fna'**	*Fulvimonas soli* DSM 14263	31.2	[27.8 - 34.7]	24.2	[21.9 - 26.7]	28.5	[25.6 - 31.6]	5.03
**'Ga-4.fna'**	*Frateuria defendens* DHoT	26.1	[22.8 - 29.7]	23.3	[21.1 - 25.8]	24.4	[21.6 - 27.6]	3.23
**'Ga-4.fna'**	*Rhodanobacter glycinis* MO64	26.4	[23.1 - 30.1]	23.0	[20.7 - 25.4]	24.6	[21.8 - 27.7]	1.74
**'Ga-4.fna'**	*Frateuria flava* MAH- 13T	25.8	[22.4 - 29.4]	22.9	[20.6 - 25.4]	24.1	[21.3 - 27.2]	1.38
**'Ga-4.fna'**	*Dyella solisilvae* DHG54	22.3	[19.0 - 25.9]	22.0	[19.8 - 24.5]	21.2	[18.5 - 24.3]	1.3
**'Ga-4.fna'**	*Dyella soli* KACC 12747	22.8	[19.5 - 26.4]	21.7	[19.4 - 24.1]	21.6	[18.8 - 24.7]	1.64
**'Ga-4.fna'**	*Dyella amyloliquefaciens* DHC06	21.4	[18.2 - 25.1]	21.6	[19.4 - 24.1]	20.5	[17.8 - 23.6]	2.1
**'Ga-4.fna'**	*Dyella japonica* DSM 16301	19.8	[16.7 - 23.4]	21.4	[19.2 - 23.8]	19.2	[16.5 - 22.2]	2.58
**'Ga-4.fna'**	*Dyella tabacisoli* L4-6	16.3	[13.4 - 19.8]	21.1	[18.9 - 23.5]	16.3	[13.7 - 19.2]	6.21

TYPE (STRAIN) GENOME SERVER

JOB SUMMARY

PRINT DATE:	2022-01-12 14:46:44 +0100
JOB ID:	f5f7d7b3-7b69-4a95-854d-e735ad2cd626
RESULT PAGE:	https://tygs.dsmz.de/user_results/show?guid=f5f7d7b3-7b69-4a95-854d-e735ad2cd626

Table 1: Phylogenies

Publication-ready versions of both the genome-scale GBDP tree and the 16S rRNA gene sequence tree can be customized and exported either in SVG (vector graphic) or PNG format from within the phylogeny viewers in your TYGS result page. For publications the **SVG format is recommended** because it is lossless, always keeps its high resolution and can also be easily converted to other popular formats such as PDF or EPS. Please follow the link provided above!

Table 2: Identification

The below list contains the result of the TYGS species identification routine.

Explanation of remarks that might occur in the below table:

remark [R1]: The TYGS type strain database is automatically updated on an almost daily basis. However, if a particular type strain genome is not available in the TYGS database, this can have several reasons which are detailed in the FAQ. You can request an extended 16S rRNA gene analysis via the 16S tree viewer found in your result page to detect **not yet genome-sequenced** type strains relevant for your study.

remark [R2]: > 70% dDDH value (formula d_4) and (almost) minimal dDDH values for gene-content formulae d_0 and d_6 indicate a potentially unreliable identification result and should thus be checked via the 16S rRNA gene sequence similarity. Such strong deviations can, in principle, be caused by sequence contamination.

remark [R3]: G+C content difference of > 1 % indicates a potentially unreliable identification result because within species G+C content varies no more than 1 %, if computed from genome sequences (PMID: 24505073).

Strain	Conclusion	Identification result	Remark
**'JalesW-56FNA'**	potential new species		see [R1]

Table 3: Pairwise comparisons of user genomes vs. type-strain genomes

The following table contains the pairwise dDDH values between your user genomes and the selected type-strain genomes. The dDDH values are provided along with their confidence intervals (C.I.) for the three different GBDP formulas:

- formula d_0 (a.k.a. GGDC formula 1): length of all HSPs divided by total genome length
- formula d_4 (a.k.a. GGDC formula 2): sum of all identities found in HSPs divided by overall HSP length
- formula d_6 (a.k.a. GGDC formula 3): sum of all identities found in HSPs divided by total genome length

Note: Formula d_4 is independent of genome length and is thus robust against the use of incomplete draft genomes. For other reasons for preferring formula d_4, see the FAQ.

Query	Subject	d_0	C.I. d_0	d_4	C.I. d_4	d_6	C.I. d_6	Diff. G+C Percent
**'JalesW-56FNA.fna'**	*Undibacterium terreum* CGMCC 1.10998	14.1	[11.3 - 17.5]	21.2	[18.9 - 23.6]	14.3	[11.9 - 17.2]	2.38
**'JalesW-56FNA.fna'**	*Undibacterium flavidum* LX15W	13.4	[10.7 - 16.7]	20.9	[18.6 - 23.3]	13.7	[11.3 - 16.5]	6.57
**'JalesW-56FNA.fna'**	*Undibacterium seohonense* KACC 16656	13.3	[10.6 - 16.7]	20.9	[18.7 - 23.3]	13.7	[11.3 - 16.5]	6.14
**'JalesW-56FNA.fna'**	*Undibacterium amnicola* KCTC 52442	13.4	[10.6 - 16.7]	20.7	[18.4 - 23.1]	13.7	[11.3 - 16.5]	7.15
**'JalesW-56FNA.fna'**	*Solimicrobium silvestre* S20-91	13.0	[10.3 - 16.3]	20.6	[18.4 - 23.0]	13.3	[11.0 - 16.1]	5.76
**'JalesW-56FNA.fna'**	*Herbaspirillum rhizosphaerae* UMS-37	13.3	[10.6 - 16.7]	20.6	[18.4 - 23.0]	13.7	[11.3 - 16.5]	7.68
**'JalesW-56FNA.fna'**	*Undibacterium piscinae* S11R28	14.8	[12.0 - 18.3]	20.4	[18.2 - 22.8]	15.0	[12.5 - 17.9]	1.0
**'JalesW-56FNA.fna'**	*Undibacterium pigrum* DSM 19792	14.3	[11.5 - 17.7]	20.4	[18.2 - 22.8]	14.5	[12.1 - 17.4]	1.78
**'JalesW-56FNA.fna'**	*Herbaspirillum rubrisubalbicans* NBRC 102523	13.0	[10.2 - 16.2]	20.3	[18.1 - 22.8]	13.3	[11.0 - 16.1]	9.11
**'JalesW-56FNA.fna'**	*Undibacterium umbellatum* NL8W	14.3	[11.5 - 17.7]	20.2	[18.0 - 22.6]	14.5	[12.1 - 17.4]	2.01
**'JalesW-56FNA.fna'**	*Undibacterium crateris* B2R-29	14.3	[11.5 - 17.7]	20.2	[18.0 - 22.6]	14.5	[12.1 - 17.3]	0.76
**'JalesW-56FNA.fna'**	*Undibacterium rivi* FT147W	14.7	[11.8 - 18.1]	20.1	[17.9 - 22.6]	14.8	[12.4 - 17.7]	2.49
**'JalesW-56FNA.fna'**	*Undibacterium aquatile* CCTCC AB 2015119	14.8	[11.9 - 18.2]	20.1	[17.8 - 22.5]	14.9	[12.4 - 17.8]	2.51
**'JalesW-56FNA.fna'**	*Herbaspirillum robiniae* HZ10	13.0	[10.3 - 16.3]	20.0	[17.8 - 22.4]	13.3	[11.0 - 16.1]	12.43
**'JalesW-56FNA.fna'**	*Undibacterium jejuense* KACC 12607	14.1	[11.2 - 17.4]	20.0	[17.8 - 22.4]	14.3	[11.9 - 17.1]	6.25
**'JalesW-56FNA.fna'**	*Minibacterium massiliensis* Marseille	13.5	[10.8 - 16.9]	19.9	[17.7 - 22.4]	13.8	[11.4 - 16.6]	1.79
**'JalesW-56FNA.fna'**	*Duganella fentianensis* FT93W	13.2	[10.5 - 16.5]	19.8	[17.6 - 22.2]	13.6	[11.2 - 16.3]	8.5
**'JalesW-56FNA.fna'**	*Undibacterium squillarum* KCTC 23917	13.5	[10.7 - 16.8]	19.6	[17.4 - 22.0]	13.8	[11.4 - 16.6]	0.05
