# Supplementary material for: Genome mining to unravel potential metabolic pathways linked to gallium bioleaching ability of bacterial mine isolates
Source: Front Microbiol. 2022 Sep 13;13:970147. doi: 10.3389/fmicb.2022.970147 (PMC9518604; doi:10.3389/fmicb.2022.970147)
Supplement: Supplementary file 4 [file Table_3.DOCX]

Supplementary Material

**Genome mining to unravel potential metabolic pathways linked to gallium bioleaching ability of bacterial mine isolates**

**Ana Paula Chung^1*^, Romeu Francisco^1^, Paula V. Morais^1^, Rita Branco^1^**

^1^University of Coimbra, Centre for Mechanical Engineering, Materials and Processes, Department of Life Sciences, Calçada Martim de Freitas, 3000-456 Coimbra, Portugal.


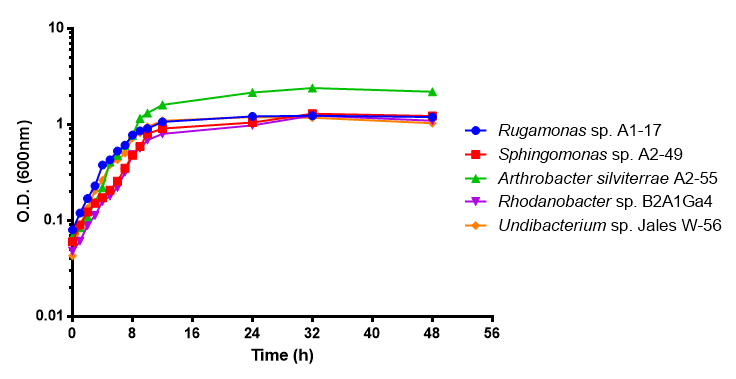


**Figure S1.** Growth curves of the 5 bacterial strains used in this study, showing the different growth phases (late exponential, 8h; stationary, 24h, and late stationary, 48h). All bacterial strain were cultured in 100 ml of mR2Ab medium, pH=6.0, at 25°C, and on rotary shaker with 140 rpm.

**Table S1** - Two-factor PERMANOVA of Ga leaching from GaAs and GaN in batch cultures, determined after 7, 14 and 21 days of incubation. Results obtained from group of replicates. Factors analysed: 1) Incubation time: D7: 7 days of incubation, D14: 14 days of incubation, D21: 21 days of incubation; 2) Strains used: Strain1: *Arthrobacter silviterrae* A2-55, Strain2: *Rhodanobacter* sp. B2A1Ga4, Strain3: *Sphingomonas* sp. A2-49, Strain4: *Undibacterium* sp. Jales W-56, Strain5: *Rugamonas* sp. A1-17. Significant differences (*p*<0.05) are marked in bold red.

| **Two-factor PERMANOVA** | **GaAs in culture** | **GaN in culture** |
| --- | --- | --- |
|  | P(MC) | P(MC) |
| **Main** |  |  |
| D7-14-21 | **0.002** | **0.001** |
| Strains | **0.001** | 0.081 |
| [D7-14-21]x[Strains] | 0.261 | **0.015** |
| **Pair-wise tests** |  |  |
| D7-D14 | **0.006** | **0.004** |
| D7-D21 | **0.001** | **0.001** |
| D14-D21 | **0.023** | **0.005** |
| Strain1–Strain2 | **0.003** | 0.070 |
| Strain1-Strain3 | **0.001** | **0.022** |
| Strain1-Strain4 | 0.293 | **0.006** |
| Strain1-Strain5 | 0.058 | 0.098 |
| Strain2-Strain3 | **0.003** | 0.371 |
| Strain2-Strain4 | **0.002** | 0.195 |
| Strain2-Strain5 | **0.026** | 0.622 |
| Strain3-Strain4 | **0.002** | 0.507 |
| Strain3-Strain5 | **0.001** | 0.952 |
| Strain4-Strain5 | **0.036** | 0.507 |
| **Interactions** |  |  |
| **[D7-14-21]x[Strains]**  **Factor D7-14-21** |  |  |
| **For Strain1** |  |  |
| D7-D14 | 0.428 | **0.009** |
| D7-D21 | 0.117 | **0.012** |
| D14-D21 | 0.354 | 0.765 |
| **For Strain2** |  |  |
| D7-D14 | **0.004** | 0.870 |
| D7-D21 | **0.010** | **0.047** |
| D14-D21 | **0.046** | **0.019** |
| **For Strain3** |  |  |
| D7-D14 | **0.045** | **0.018** |
| D7-D21 | **0.049** | 0.201 |
| D14-D21 | 0.333 | 0.704 |
| **For Strain4** |  |  |
| D7-D14 | 0.392 | 0.907 |
| D7-D21 | 0.270 | 0.286 |
| D14-D21 | 0.279 | **0.039** |
| **For Strain5** |  |  |
| D7-D14 | 0.060 | 0.321 |
| D7-D21 | **0.007** | 0.198 |
| D14-D21 | 0.372 | 0.396 |
| **[D7-14-21]x[Strains]**  **Factor Strains** |  |  |
| **For D7** |  |  |
| Strain1–Strain2 | 0.095 | 0.116 |
| Strain1-Strain3 | **0.041** | **0.008** |
| Strain1-Strain4 | 0.481 | 0.056 |
| Strain1-Strain5 | 0.800 | 0.057 |
| Strain2-Strain3 | 0.061 | 0.615 |
| Strain2-Strain4 | 0.225 | 0.316 |
| Strain2-Strain5 | **0.013** | 0.714 |
| Strain3-Strain4 | 0.141 | 0.343 |
| Strain3-Strain5 | **0.010** | 0.939 |
| Strain4-Strain5 | 0.444 | 0.412 |
| **For D14** |  |  |
| Strain1–Strain2 | 0.114 | **0.034** |
| Strain1-Strain3 | **0.039** | 0.876 |
| Strain1-Strain4 | 0.603 | 0.378 |
| Strain1-Strain5 | 0.259 | 0.541 |
| Strain2-Strain3 | **0.049** | **0.013** |
| Strain2-Strain4 | **0.011** | **0.027** |
| Strain2-Strain5 | 0.607 | 0.150 |
| Strain3-Strain4 | **0.008** | 0.209 |
| Strain3-Strain5 | 0.143 | 0.441 |
| Strain4-Strain5 | 0.090 | 0.929 |
| **For D21** |  |  |
| Strain1–Strain2 | 0.069 | 0.067 |
| Strain1-Strain3 | 0.104 | 0.585 |
| Strain1-Strain4 | 0.570 | 0.135 |
| Strain1-Strain5 | 0.194 | 0.472 |
| Strain2-Strain3 | 0.224 | 0.239 |
| Strain2-Strain4 | **0.044** | 0.142 |
| Strain2-Strain5 | 0.147 | 0.407 |
| Strain3-Strain4 | 0.074 | 0.753 |
| Strain3-Strain5 | 0.270 | 0.809 |
| Strain4-Strain5 | 0.110 | 0.910 |

P(MC): significance after Montecarlo correction

**Table S2** - Three-factor PERMANOVA of Ga leaching from GaAs or GaN by bacterial cultures or spent medium at different growth phases. Results obtained from group of replicates. Factors analysed: 1) Growth phase: E: late exponential (8h), S: stationary (24h), L: late stationary (48h); 2) Incubation time: D7: 7 days of incubation, D14: 14 days of incubation, D21: 21 days of incubation; 3) Strains used: Strain1: *Arthrobacter silviterrae* A2-55, Strain2: *Rhodanobacter* sp. B2A1Ga4, Strain3: *Sphingomonas* sp. A2-49, Strain4: *Undibacterium* sp. Jales W-56, Strain5: *Rugamonas* sp. A1-17. Significant differences (*p*<0.05) are marked in bold red.

| **Three-factor PERMANOVA** | **Culture GaAs** | **Culture GaN** | **Spent GaAs** | **Spent GaN** |
| --- | --- | --- | --- | --- |
|  | P(MC) | P(MC) | P(MC) | P(MC) |
| **Main** |  |  |  |  |
| E-S-L | 0.620 | **0.001** | **0.001** | 0.801 |
| D7-14-21 | **0.001** | **0.001** | **0.001** | **0.001** |
| Strains | 0.101 | **0.001** | **0.001** | **0.001** |
| [E-S-L]x[D7-14-21] | 0.081 | 0.052 | 0.287 | **0.044** |
| [E-S-L]x[Strains] | 0.113 | **0.040** | **0.001** | **0.001** |
| [D7-14-21]x[Strains] | **0.002** | **0.001** | **0.001** | 0.055 |
| [E-S-L]x[D7-14-21]x[Strains] | **0.004** | 0.460 | **0.004** | 0.160 |
| **Pair-wise tests** |  |  |  |  |
| E-S | 0.517 | **0.002** |  |  |
| E-L | 0.713 | **0.002** |  |  |
| S-L | 0.540 | 0.244 | **0.001** | 0.780 |
| D7-D14 | **0.014** | 0.064 | **0.001** | **0.001** |
| D7-D21 | **0.002** | **0.001** | **0.001** | **0.001** |
| D14-D21 | **0.001** | **0.001** | **0.001** | **0.001** |
| Strain1–Strain2 | 0.447 | 0.501 | 0.100 | 0.347 |
| Strain1-Strain3 | 0.265 | 0.371 | **0.001** | **0.001** |
| Strain1-Strain4 | 0.743 | **0.001** | **0.001** | 0.378 |
| Strain1-Strain5 | 0.205 | **0.001** | **0.001** | 0.690 |
| Strain2-Strain3 | 0.176 | 0.220 | **0.001** | **0.002** |
| Strain2-Strain4 | 0.244 | **0.001** | **0.004** | 0.886 |
| Strain2-Strain5 | 0.143 | **0.001** | **0.014** | 0.234 |
| Strain3-Strain4 | 0.324 | **0.001** | 0.122 | **0.001** |
| Strain3-Strain5 | 0.289 | **0.001** | **0.003** | **0.001** |
| Strain4-Strain5 | 0.198 | 0.387 | 0.149 | 0.240 |
| **Interactions** |  |  |  |  |
| **[E-S-L]x[D7-14-21]**  **Factor E-S-L** |  |  |  |  |
| **For D7** |  |  |  |  |
| E-S | 0.143 | 0.094 |  |  |
| E-L | 0.383 | 0.083 |  |  |
| S-L | 0.215 | 0.619 | 0.393 | 0.080 |
| **For D14** |  |  |  |  |
| E-S | 0.535 | **0.036** |  |  |
| E-L | 0.829 | **0.006** |  |  |
| S-L | 0.436 | 0.122 | **0.009** | 0.503 |
| **For D21** |  |  |  |  |
| E-S | 0.249 | **0.001** |  |  |
| E-L | 0.294 | **0.020** |  |  |
| S-L | 0.575 | 0.382 | **0.003** | 0.096 |
| **[E-S-L]x[D7-14-21]**  **Factor D7-14-21** |  |  |  |  |
| **For E** |  |  |  |  |
| D7-D14 | **0.019** | 0.454 |  |  |
| D7-D21 | **0.002** | **0.001** |  |  |
| D14-D21 | **0.001** | **0.001** |  |  |
| **For S** |  |  |  |  |
| D7-D14 | **0.006** | 0.327 | **0.001** | **0.021** |
| D7-D21 | **0.003** | **0.001** | **0.001** | **0.001** |
| D14-D21 | **0.007** | **0.001** | **0.002** | 0.056 |
| **For L** |  |  |  |  |
| D7-D14 | **0.026** | 0.169 | **0.001** | **0.003** |
| D7-D21 | **0.002** | **0.001** | **0.001** | **0.001** |
| D14-D21 | **0.002** | **0.001** | **0.001** | **0.004** |
| **[E-S-L]x[Strains]**  **Factor E-S-L** |  |  |  |  |
| **For Strain1** |  |  |  |  |
| E-S | 0.411 | 0.096 |  |  |
| E-L | 0.368 | 0.064 |  |  |
| S-L | 0.433 | 0.142 | 0.131 | 0.388 |
| **For Strain2** |  |  |  |  |
| E-S | 0.709 | 0.195 |  |  |
| E-L | 0.547 | 0.086 |  |  |
| S-L | 0.318 | 0.572 | **0.003** | 0.102 |
| **For Strain3** |  |  |  |  |
| E-S | 0.275 | 0.922 |  |  |
| E-L | 0.410 | 0.838 |  |  |
| S-L | 0.293 | 0.702 | **0.016** | 0.178 |
| **For Strain4** |  |  |  |  |
| E-S | 0.181 | **0.006** |  |  |
| E-L | 0.207 | **0.002** |  |  |
| S-L | 0.192 | 0.360 | 0.189 | **0.004** |
| **For Strain5** |  |  |  |  |
| E-S | 0.236 | 0.098 |  |  |
| E-L | 0.497 | 0.087 |  |  |
| S-L | 0.256 | 0.703 | 0.148 | **0.033** |
| **[E-S-L]x[Strains]**  **Factor Strains** |  |  |  |  |
| **For E** |  |  |  |  |
| Strain1–Strain2 | 0.368 | 0.342 |  |  |
| Strain1-Strain3 | 0.404 | 0.232 |  |  |
| Strain1-Strain4 | 0.512 | **0.024** |  |  |
| Strain1-Strain5 | 0.369 | **0.007** |  |  |
| Strain2-Strain3 | 0.272 | 0.727 |  |  |
| Strain2-Strain4 | 0.210 | **0.018** |  |  |
| Strain2-Strain5 | 0.165 | **0.001** |  |  |
| Strain3-Strain4 | 0.324 | 0.070 |  |  |
| Strain3-Strain5 | 0.367 | **0.004** |  |  |
| Strain4-Strain5 | 0.215 | **0.041** |  |  |
| **For S** |  |  |  |  |
| Strain1–Strain2 | 0.327 | 0.631 | **0.007** | 0.312 |
| Strain1-Strain3 | 0.210 | 0.692 | **0.001** | **0.009** |
| Strain1-Strain4 | 0.203 | **0.001** | **0.007** | 0.234 |
| Strain1-Strain5 | 0.139 | **0.001** | **0.003** | 0.122 |
| Strain2-Strain3 | 0.193 | 0.426 | 0.104 | 0.109 |
| Strain2-Strain4 | 0.416 | **0.006** | 0.250 | **0.033** |
| Strain2-Strain5 | 0.118 | **0.004** | 0.699 | **0.024** |
| Strain3-Strain4 | 0.170 | **0.003** | 0.280 | **0.001** |
| Strain3-Strain5 | 0.186 | **0.001** | 0.083 | **0.001** |
| Strain4-Strain5 | 0.119 | 0.431 | 0.318 | 0.606 |
| **For L** |  |  |  |  |
| Strain1–Strain2 | 0.507 | 0.777 | **0.026** | 0.842 |
| Strain1-Strain3 | 0.419 | **0.040** | **0.006** | **0.016** |
| Strain1-Strain4 | 0.458 | **0.002** | **0.025** | **0.035** |
| Strain1-Strain5 | 0.279 | **0.007** | 0.176 | 0.143 |
| Strain2-Strain3 | 0.205 | 0.112 | **0.001** | **0.012** |
| Strain2-Strain4 | 0.237 | **0.004** | **0.002** | **0.028** |
| Strain2-Strain5 | 0.116 | **0.009** | **0.005** | 0.183 |
| Strain3-Strain4 | 0.442 | **0.001** | **0.029** | 0.150 |
| Strain3-Strain5 | 0.285 | **0.001** | **0.005** | **0.045** |
| Strain4-Strain5 | 0.320 | 0.324 | 0.279 | 0.287 |
| **[D7-14-21]x[Strains]**  **Factor D7-14-21** |  |  |  |  |
| **For Strain1** |  |  |  |  |
| D7-D14 | 0.051 | 0.950 | **0.002** | **0.006** |
| D7-D21 | **0.012** | **0.001** | **0.002** | **0.005** |
| D14-D21 | **0.006** | **0.001** | **0.005** | 0.875 |
| **For Strain2** |  |  |  |  |
| D7-D14 | 0.087 | 0.360 | **0.018** | **0.041** |
| D7-D21 | **0.008** | **0.001** | **0.010** | **0.035** |
| D14-D21 | **0.002** | **0.001** | 0.167 | 0.757 |
| **For Strain3** |  |  |  |  |
| D7-D14 | 0.092 | 0.360 | **0.013** | 0.652 |
| D7-D21 | **0.016** | **0.001** | **0.001** | **0.021** |
| D14-D21 | **0.006** | **0.001** | **0.011** | 0.063 |
| **For Strain4** |  |  |  |  |
| D7-D14 | **0.030** | **0.008** | 0.107 | 0.278 |
| D7-D21 | **0.009** | **0.001** | **0.002** | **0.006** |
| D14-D21 | **0.013** | **0.001** | **0.001** | **0.006** |
| **For Strain5** |  |  |  |  |
| D7-D14 | **0.033** | **0.039** | **0.002** | 0.329 |
| D7-D21 | **0.002** | **0.001** | **0.001** | **0.010** |
| D14-D21 | **0.004** | **0.001** | **0.031** | **0.020** |
| **[D7-14-21]x[Strains]**  **Factor Strains** |  |  |  |  |
| **For D7** |  |  |  |  |
| Strain1–Strain2 | 0.105 | 0.435 | 0.096 | 0.740 |
| Strain1-Strain3 | 0.725 | 0.750 | 0.410 | 0.097 |
| Strain1-Strain4 | 0.266 | 0.050 | 0.555 | 0.217 |
| Strain1-Strain5 | 0.696 | **0.012** | 0.457 | 0.061 |
| Strain2-Strain3 | 0.068 | 0.175 | **0.047** | 0.082 |
| Strain2-Strain4 | 0.212 | 0.133 | 0.200 | 0.375 |
| Strain2-Strain5 | **0.005** | **0.026** | 0.146 | 0.107 |
| Strain3-Strain4 | 0.316 | **0.007** | 0.176 | 0.056 |
| Strain3-Strain5 | 0.705 | **0.003** | 0.089 | **0.027** |
| Strain4-Strain5 | 0.079 | 0.358 | 0.960 | 0.491 |
| **For D14** |  |  |  |  |
| Strain1–Strain2 | 0.774 | 0.673 | 0.149 | 0.465 |
| Strain1-Strain3 | 0.051 | 0.550 | **0.003** | **0.009** |
| Strain1-Strain4 | 0.407 | **0.001** | **0.001** | **0.045** |
| Strain1-Strain5 | **0.014** | **0.001** | **0.003** | 0.205 |
| Strain2-Strain3 | 0.053 | 0.758 | **0.001** | **0.030** |
| Strain2-Strain4 | 0.199 | **0.001** | **0.002** | 0.251 |
| Strain2-Strain5 | **0.012** | **0.001** | **0.008** | 0.648 |
| Strain3-Strain4 | 0.124 | **0.001** | 0.798 | **0.038** |
| Strain3-Strain5 | 0.246 | **0.001** | **0.037** | **0.028** |
| Strain4-Strain5 | 0.108 | 0.357 | **0.044** | 0.291 |
| **For D21** |  |  |  |  |
| Strain1–Strain2 | 0.476 | 0.908 | **0.026** | 0.499 |
| Strain1-Strain3 | 0.243 | 0.250 | **0.001** | 0.077 |
| Strain1-Strain4 | 0.330 | **0.002** | **0.011** | 0.676 |
| Strain1-Strain5 | **0.018** | **0.005** | **0.023** | 0.430 |
| Strain2-Strain3 | 0.120 | 0.145 | **0.027** | 0.387 |
| Strain2-Strain4 | 0.612 | **0.001** | 0.320 | 0.504 |
| Strain2-Strain5 | **0.028** | **0.001** | 0.475 | 0.252 |
| Strain3-Strain4 | **0.001** | **0.002** | 0.055 | **0.048** |
| Strain3-Strain5 | **0.024** | **0.001** | 0.102 | **0.014** |
| Strain4-Strain5 | **0.009** | 0.718 | 0.914 | 0.651 |
| **[E-S-L]x[D7-14-21]x[Strains]**  **Factor E-S-L** |  |  |  |  |
| **For D7 and Strain1** |  |  |  |  |
| E-S | **0.029** | 0.604 |  |  |
| E-L | 0.513 | 0.600 |  |  |
| S-L | 0.184 | 0.182 | 0.432 | 0.294 |
| **For D7 and Strain2** |  |  |  |  |
| E-S | 0.060 | 0.442 |  |  |
| E-L | **0.034** | 0.455 |  |  |
| S-L | 0.603 | 0.913 | 0.197 | 0.742 |
| **For D7 and Strain3** |  |  |  |  |
| E-S | **0.019** | 0.626 |  |  |
| E-L | **0.029** | 0.658 |  |  |
| S-L | 0.532 | 0.333 | 0.051 | 0.677 |
| **For D7 and Strain4** |  |  |  |  |
| E-S | 0.259 | 0.159 |  |  |
| E-L | 0.227 | 0.098 |  |  |
| S-L | 0.077 | 0.714 | 0.903 | 0.271 |
| **For D7 and Strain5** |  |  |  |  |
| E-S | 0.606 | 0.681 |  |  |
| E-L | 0.905 | 0.568 |  |  |
| S-L | 0.761 | 0.812 | 0.272 | 0.367 |
| **For D14 and Strain1** |  |  |  |  |
| E-S | 0.210 | 0.099 |  |  |
| E-L | 0.161 | 0.131 |  |  |
| S-L | 0.586 | 0.745 | 0.424 | 0.455 |
| **For D14 and Strain2** |  |  |  |  |
| E-S | 0.671 | 0.544 |  |  |
| E-L | 0.291 | 0.186 |  |  |
| S-L | 0.576 | 0.365 | **0.029** | 0.374 |
| **For D14 and Strain3** |  |  |  |  |
| E-S | 0.130 | 0.556 |  |  |
| E-L | 0.704 | 0.955 |  |  |
| S-L | 0.209 | 0.428 | 0.558 | 0.891 |
| **For D14 and Strain4** |  |  |  |  |
| E-S | **0.035** | 0.111 |  |  |
| E-L | 0.154 | **0.025** |  |  |
| S-L | **0.021** | 0.249 | 0.125 | **0.014** |
| **For D14 and Strain5** |  |  |  |  |
| E-S | 0.300 | 0.290 |  |  |
| E-L | 0.707 | 0.295 |  |  |
| S-L | 0.497 | 0.787 | 0.451 | 0.239 |
| **For D21 and Strain1** |  |  |  |  |
| E-S | **0.035** | 0.524 |  |  |
| E-L | **0.033** | 0.269 |  |  |
| S-L | 0.132 | 0.732 | 0.057 | 0.759 |
| **For D21 and Strain2** |  |  |  |  |
| E-S | 0.682 | 0.087 |  |  |
| E-L | 0.100 | 0.248 |  |  |
| S-L | 0.074 | 0.101 | **0.022** | 0.202 |
| **For D21 and Strain3** |  |  |  |  |
| E-S | 0.140 | 0.576 |  |  |
| E-L | 0.132 | 0.865 |  |  |
| S-L | 0.610 | 0.741 | 0.076 | **0.046** |
| **For D21 and Strain4** |  |  |  |  |
| E-S | 0.283 | **0.037** |  |  |
| E-L | 0.187 | 0.124 |  |  |
| S-L | 0.666 | 0.185 | 0.548 | 0.100 |
| **For D21 and Strain5** |  |  |  |  |
| E-S | 0.582 | 0.117 |  |  |
| E-L | 0.271 | 0.169 |  |  |
| S-L | 0.235 | 0.811 | 0.428 | 0.126 |
| **[E-S-L]x[D7-14-21]x[Strains]**  **Factor D7-14-21** |  |  |  |  |
| **For E and Strain1** |  |  |  |  |
| D7-D14 | **0.016** | 0.729 |  |  |
| D7-D21 | **0.011** | 0.086 |  |  |
| D14-D21 | **0.005** | **0.031** |  |  |
| **For E and Strain2** |  |  |  |  |
| D7-D14 | **0.037** | 0.508 |  |  |
| D7-D21 | **0.005** | **0.020** |  |  |
| D14-D21 | **0.025** | **0.020** |  |  |
| **For E and Strain3** |  |  |  |  |
| D7-D14 | **0.018** | 0.580 |  |  |
| D7-D21 | **0.001** | **0.036** |  |  |
| D14-D21 | **0.015** | **0.048** |  |  |
| **For E and Strain4** |  |  |  |  |
| D7-D14 | 0.078 | 0.085 |  |  |
| D7-D21 | **0.011** | **0.018** |  |  |
| D14-D21 | **0.003** | **0.012** |  |  |
| **For E and Strain5** |  |  |  |  |
| D7-D14 | 0.089 | 0.371 |  |  |
| D7-D21 | **0.005** | **0.007** |  |  |
| D14-D21 | **0.047** | **0.017** |  |  |
| **For S and Strain1** |  |  |  |  |
| D7-D14 | **0.028** | 0.084 | **0.024** | 0.171 |
| D7-D21 | **0.001** | **0.003** | **0.021** | 0.077 |
| D14-D21 | **0.014** | **0.022** | **0.007** | 0.713 |
| **For S and Strain2** |  |  |  |  |
| D7-D14 | 0.174 | 0.539 | 0.107 | 0.434 |
| D7-D21 | **0.001** | **0.047** | 0.122 | 0.534 |
| D14-D21 | **0.021** | **0.009** | 0.252 | 0.916 |
| **For S and Strain3** |  |  |  |  |
| D7-D14 | **0.022** | 0.597 | 0.504 | 0.857 |
| D7-D21 | **0.003** | 0.051 | 0.144 | 0.491 |
| D14-D21 | **0.006** | **0.022** | 0.197 | 0.721 |
| **For S and Strain4** |  |  |  |  |
| D7-D14 | **0.043** | 0.347 | 0.778 | 0.090 |
| D7-D21 | **0.005** | **0.016** | **0.041** | **0.022** |
| D14-D21 | **0.004** | **0.018** | **0.016** | **0.014** |
| **For S and Strain5** |  |  |  |  |
| D7-D14 | 0.607 | 0.151 | **0.011** | 0.309 |
| D7-D21 | **0.032** | **0.006** | **0.023** | **0.031** |
| D14-D21 | 0.055 | **0.003** | 0.214 | **0.025** |
| **For L and Strain1** |  |  |  |  |
| D7-D14 | 0.080 | 0.866 | **0.032** | **0.037** |
| D7-D21 | **0.021** | 0.089 | **0.010** | **0.025** |
| D14-D21 | **0.030** | **0.019** | 0.205 | 0.771 |
| **For L and Strain2** |  |  |  |  |
| D7-D14 | 0.121 | 0.809 | 0.149 | **0.048** |
| D7-D21 | **0.001** | 0.115 | 0.065 | **0.020** |
| D14-D21 | **0.006** | **0.007** | 0.193 | 0.403 |
| **For L and Strain3** |  |  |  |  |
| D7-D14 | 0.146 | 0.182 | **0.026** | 0.568 |
| D7-D21 | **0.003** | **0.016** | **0.012** | **0.025** |
| D14-D21 | **0.009** | **0.040** | 0.052 | **0.045** |
| **For L and Strain4** |  |  |  |  |
| D7-D14 | 0.078 | 0.137 | **0.039** | 0.845 |
| D7-D21 | **0.010** | **0.033** | **0.012** | 0.158 |
| D14-D21 | **0.003** | 0.057 | 0.052 | 0.138 |
| **For L and Strain5** |  |  |  |  |
| D7-D14 | 0.383 | 0.298 | **0.048** | 0.658 |
| D7-D21 | **0.025** | **0.027** | **0.017** | 0.152 |
| D14-D21 | **0.049** | **0.018** | 0.074 | 0.284 |
| **[E-S-L]x[D7-14-21]x[Strains]**  **Factor Strains** |  |  |  |  |
| **For E and D7** |  |  |  |  |
| Strain1–Strain2 | **0.022** | 0.579 |  |  |
| Strain1-Strain3 | 0.785 | 0.734 |  |  |
| Strain1-Strain4 | **0.044** | 0.498 |  |  |
| Strain1-Strain5 | 0.192 | 0.217 |  |  |
| Strain2-Strain3 | **0.005** | 0.741 |  |  |
| Strain2-Strain4 | 0.350 | 0.779 |  |  |
| Strain2-Strain5 | **0.010** | 0.094 |  |  |
| Strain3-Strain4 | **0.016** | 0.608 |  |  |
| Strain3-Strain5 | 0.057 | 0.099 |  |  |
| Strain4-Strain5 | **0.042** | 0.125 |  |  |
| **For E and D14** |  |  |  |  |
| Strain1–Strain2 | 0.131 | 0.466 |  |  |
| Strain1-Strain3 | 0.075 | 0.202 |  |  |
| Strain1-Strain4 | 0.214 | **0.046** |  |  |
| Strain1-Strain5 | 0.103 | **0.039** |  |  |
| Strain2-Strain3 | **0.045** | 0.380 |  |  |
| Strain2-Strain4 | 0.092 | **0.047** |  |  |
| Strain2-Strain5 | 0.059 | 0.055 |  |  |
| Strain3-Strain4 | 0.116 | 0.160 |  |  |
| Strain3-Strain5 | 0.837 | 0.138 |  |  |
| Strain4-Strain5 | 0.150 | 0.499 |  |  |
| **For E and D21** |  |  |  |  |
| Strain1–Strain2 | **0.014** | 0.862 |  |  |
| Strain1-Strain3 | 0.884 | 0.805 |  |  |
| Strain1-Strain4 | 0.058 | 0.208 |  |  |
| Strain1-Strain5 | 0.051 | 0.110 |  |  |
| Strain2-Strain3 | **0.014** | 0.631 |  |  |
| Strain2-Strain4 | 0.078 | 0.106 |  |  |
| Strain2-Strain5 | **0.013** | **0.045** |  |  |
| Strain3-Strain4 | 0.063 | 0.144 |  |  |
| Strain3-Strain5 | **0.046** | 0.066 |  |  |
| Strain4-Strain5 | **0.029** | 0.514 |  |  |
| **For S and D7** |  |  |  |  |
| Strain1–Strain2 | **0.034** | 0.176 | 0.555 | 0.944 |
| Strain1-Strain3 | **0.041** | 0.408 | 0.285 | 0.342 |
| Strain1-Strain4 | 0.557 | **0.028** | 0.493 | 0.115 |
| Strain1-Strain5 | 0.073 | **0.012** | 0.514 | 0.139 |
| Strain2-Strain3 | **0.008** | 0.700 | 0.690 | 0.355 |
| Strain2-Strain4 | 0.281 | 0.341 | 0.973 | 0.209 |
| Strain2-Strain5 | **0.045** | 0.326 | 0.772 | 0.196 |
| Strain3-Strain4 | 0.064 | 0.201 | 0.658 | 0.147 |
| Strain3-Strain5 | 0.186 | 0.170 | 0.208 | 0.129 |
| Strain4-Strain5 | 0.071 | 0.905 | 0.706 | 0.690 |
| **For S and D14** |  |  |  |  |
| Strain1–Strain2 | 0.883 | 0.344 | **0.009** | 0.646 |
| Strain1-Strain3 | 0.169 | 0.161 | **0.009** | 0.192 |
| Strain1-Strain4 | 0.742 | **0.040** | **0.022** | 0.866 |
| Strain1-Strain5 | **0.039** | **0.020** | **0.023** | 0.867 |
| Strain2-Strain3 | 0.260 | 0.800 | **0.030** | 0.365 |
| Strain2-Strain4 | 0.948 | **0.049** | **0.049** | 0.496 |
| Strain2-Strain5 | **0.047** | **0.044** | 0.128 | 0.450 |
| Strain3-Strain4 | 0.066 | **0.024** | 0.455 | 0.090 |
| Strain3-Strain5 | 0.086 | **0.017** | 0.122 | 0.096 |
| Strain4-Strain5 | 0.053 | 0.735 | 0.093 | 0.811 |
| **For S and D21** |  |  |  |  |
| Strain1–Strain2 | **0.045** | 0.523 | **0.002** | 0.411 |
| Strain1-Strain3 | 0.067 | 0.699 | **0.008** | 0.080 |
| Strain1-Strain4 | 0.318 | **0.047** | **0.015** | 0.267 |
| Strain1-Strain5 | 0.050 | 0.108 | 0.066 | 0.198 |
| Strain2-Strain3 | **0.020** | 0.267 | 0.195 | 0.480 |
| Strain2-Strain4 | 0.156 | **0.018** | 0.080 | 0.125 |
| Strain2-Strain5 | **0.029** | **0.047** | 0.327 | 0.126 |
| Strain3-Strain4 | 0.079 | **0.028** | 0.072 | **0.007** |
| Strain3-Strain5 | 0.058 | 0.057 | 0.183 | **0.013** |
| Strain4-Strain5 | **0.027** | 0.117 | 0.828 | 0.668 |
| **For L and D7** |  |  |  |  |
| Strain1–Strain2 | 0.133 | 0.951 | 0.122 | 0.674 |
| Strain1-Strain3 | 0.534 | 0.194 | 0.088 | 0.201 |
| Strain1-Strain4 | 0.594 | 0.341 | 0.935 | 0.817 |
| Strain1-Strain5 | 0.932 | 0.436 | 0.718 | 0.368 |
| Strain2-Strain3 | **0.024** | 0.241 | **0.027** | 0.155 |
| Strain2-Strain4 | **0.038** | 0.330 | 0.087 | 0.936 |
| Strain2-Strain5 | **0.047** | 0.375 | 0.116 | 0.466 |
| Strain3-Strain4 | 0.779 | **0.025** | 0.076 | 0.272 |
| Strain3-Strain5 | 0.375 | **0.032** | 0.053 | 0.110 |
| Strain4-Strain5 | 0.501 | 0.775 | 0.612 | 0.608 |
| **For L and D14** |  |  |  |  |
| Strain1–Strain2 | 0.349 | 0.741 | 0.301 | 0.591 |
| Strain1-Strain3 | 0.064 | 0.408 | 0.051 | 0.053 |
| Strain1-Strain4 | 0.052 | **0.023** | 0.055 | **0.043** |
| Strain1-Strain5 | 0.082 | **0.045** | 0.142 | 0.151 |
| Strain2-Strain3 | 0.095 | 0.606 | **0.032** | 0.051 |
| Strain2-Strain4 | 0.067 | **0.005** | **0.030** | **0.035** |
| Strain2-Strain5 | 0.088 | **0.033** | 0.061 | 0.213 |
| Strain3-Strain4 | 0.700 | **0.021** | 0.397 | 0.332 |
| Strain3-Strain5 | 0.422 | **0.031** | 0.207 | 0.172 |
| Strain4-Strain5 | 0.305 | 0.165 | 0.371 | 0.322 |
| **For L and D21** |  |  |  |  |
| Strain1–Strain2 | 0.172 | 0.264 | 0.318 | 0.654 |
| Strain1-Strain3 | 0.068 | 0.320 | 0.191 | 0.875 |
| Strain1-Strain4 | 0.826 | 0.069 | 0.328 | 0.360 |
| Strain1-Strain5 | **0.020** | 0.072 | 0.507 | 0.557 |
| Strain2-Strain3 | 0.562 | 0.539 | 0.112 | 0.600 |
| Strain2-Strain4 | 0.234 | **0.027** | 0.148 | 0.220 |
| Strain2-Strain5 | 0.059 | **0.032** | 0.182 | 0.370 |
| Strain3-Strain4 | 0.083 | 0.063 | 0.686 | 0.462 |
| Strain3-Strain5 | **0.046** | 0.069 | 0.355 | 0.676 |
| Strain4-Strain5 | **0.020** | 0.872 | 0.696 | 0.712 |

P(MC): significance after Montecarlo correction

**Table S3** - Percentage of Ga leached by different mixtures of amino acids after15 days

| **Amino acids mixtures** | **5 days** | | **10 days** | | **15 days** | |
| --- | --- | --- | --- | --- | --- | --- |
|  | **GaAs** | **GaN** | **GaAs** | **GaN** | **GaAs** | **GaN** |
| **Arg/ Lys/ His** | \| **16.7**±2.01 \| \| --- \| | 5.8± 0.2 | **27.2**±2.14 | 7.1±0.8 | **34.6**±0.82 | 10.3±0.45 |
| **Glu/Asp** | 7.3±0.90 | 4.2± 0.3 | 12.8±0.51 | 6.2±0.92 | 17.8±1.03 | 8.9±0.07 |
| **Tyr/Trp** | 7.1±0.71 | 3.5± 0.15 | 9.5±0.15 | 5.4±0.187 | 12.5±0.92 | 6.5±0.20 |
| **Cyst/Met** | 3.1 ±0.32 | 2.2± 0.18 | 5.2±0.4 | 3.1±0.25 | 7.08±0.55 | 4.2±0.82 |

The leaching experiments were performed in 20 ml of an aqueous mixture of amino acids (pH=8.5), with either 10 mg of GaAs or GaN, at 25°C and 150 rpm, in an orbital shaking incubator for 15 days. Four different mixtures of amino acids were used: Mixture 1 (arginine, lysine and arginine); Mixture 2 (glutamic and aspartic acid); Mixture 3 (tyrosine and tryptophan) and Mixture 4 (cysteine and methionine). All amino acids were prepared at a concentration of 15 mM with exception of tyrosine and cysteine that were prepared with a concentration of 2.7 mM and 2.2 mM respectively, due to their lower solubility.
